# One-dimensional scintillator film with benign grain boundaries for high-resolution and fast x-ray imaging

**DOI:** 10.1126/sciadv.adh1789

**Published:** 2023-07-28

**Authors:** Haodi Wu, Qian Wang, Ao Zhang, Guangda Niu, Martin Nikl, Chen Ming, Jinsong Zhu, Zhengyang Zhou, Yi-Yang Sun, Guangjun Nan, Guohao Ren, Yuntao Wu, Jiang Tang

**Affiliations:** ^1^ Wuhan National Laboratory for Optoelectronics (WNLO) and School of Optical and Electronic Information, Huazhong University of Science and Technology (HUST), Wuhan 430074, China.; ^2^ Artificial Crystal Research Center, Shanghai Institute of Ceramics, Chinese Academy of Sciences, Shanghai 201899, China.; ^3^ Optics Valley Laboratory, Hubei, 430074, China.; ^4^ Department of Optical Materials, Institute of Physics of the Czech Academy of Sciences, Cukrovarnicka 10/112, Prague 16200, Czech Republic.; ^5^ State Key Laboratory of High Performance Ceramics and Superfine Microstructure, Shanghai Institute of Ceramics, Chinese Academy of Sciences, Shanghai 201899, China.; ^6^ Department of Physics, Zhejiang Normal University, Jinhua 321004, Zhejiang, China.

## Abstract

Fast and high-resolution x-ray imaging demands scintillator films with negligible afterglow, high scintillation yield, and minimized cross-talk. However, grain boundaries (GBs) are abundant in polycrystalline scintillator film, and, for current inorganic scintillators, detrimental dangling bonds at GBs inevitably extend radioluminescence lifetime and increase nonradiative recombination loss, deteriorating afterglow and scintillation yield. Here, we demonstrate that scintillators with one-dimensional (1D) crystal structure, Cs_5_Cu_3_Cl_6_I_2_ explored here, possess benign GBs without dangling bonds, yielding nearly identical afterglow and scintillation yield for single crystals and polycrystalline films. Because of its 1D crystal structure, Cs_5_Cu_3_Cl_6_I_2_ films with desired columnar morphology are easily obtained via close space sublimation, exhibit negligible afterglow (0.1% at 10 ms) and high scintillation yield (1.2 times of CsI:Tl). We have also demonstrated fast x-ray imaging with 27 line pairs mm^−1^ resolution and frame rate up to 33 fps, surpassing most existing scintillators. We believe that the 1D scintillators can greatly boost x-ray imaging performance.

## Teaser

1D crystal structure scintillator film with benign grain boundaries achieves fast and high-resolution x-ray imaging.

## INTRODUCTION

Scintillators are indispensable for a number of technologies, including x-ray detectors used in medical imaging and nondestructive inspection (*1*), phosphor screens in night-vision systems (*2*), electron detectors in electron microscopes (*3*), and electromagnetic calorimeters in high-energy physics experiments (*4*). Polycrystalline scintillator films are used for most of these applications, especially in x-ray flat panel detector (FPD). Research in this field is largely driven by the aim of high spatial resolution and low afterglow, for tiny-spot identification and high-speed imaging (*5*, *6*). In terms of spatial resolution, in addition to high scintillation yield, scintillators with optical wave-guiding effect have been designed. The columnar structured scintillator films enjoy low scattering and inter-pixel cross-talk and have demonstrated their intrinsic advantages in high-resolution and high-pixel fill factor for x-ray FPD (*7*). The afterglow effect, which causes residual shadow and image blurring, remains as a severe challenge for scintillator films. Current approaches to reduce afterglow are mostly oriented toward the doping or codoping (e.g., Sm or Bi codoping in CsI:Tl) (*8*, *9*), as well as identification of new materials (e.g., perovskites and ceramics) (*10*). However, these approaches either have little success or at the price of a substantially decreased scintillation yield (*11*, *12*). Hence, the development of scintillator films with combined high spatial resolution and negligible afterglow for x-ray FPD remains a long-standing challenge.

**Fig. 1.**
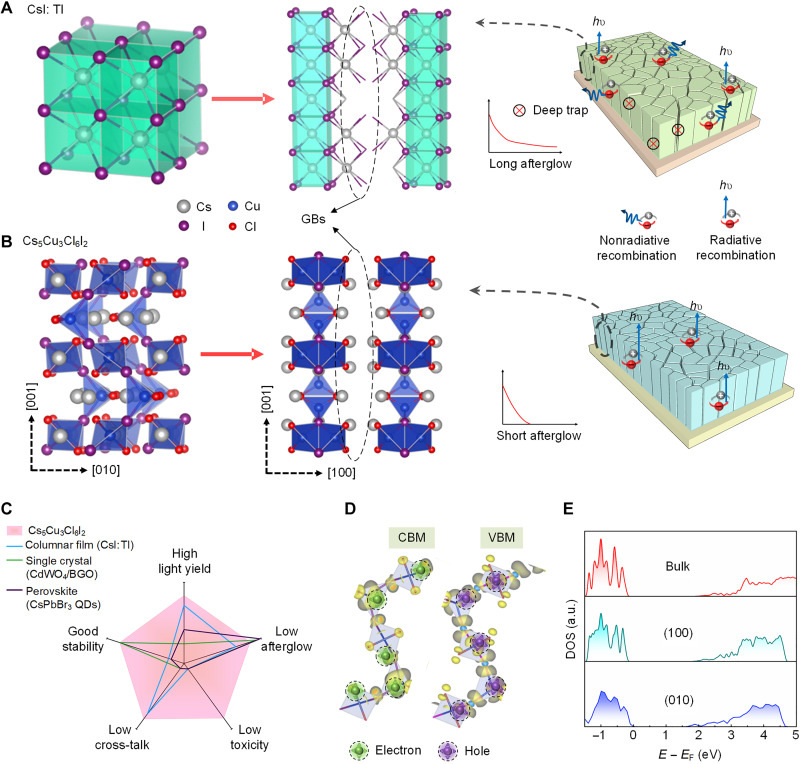
Comparison between 3D CsI:Tl and 1D Cs_5_Cu_3_Cl_6_I_2_ scintillator. (**A**) CsI:Tl has a 3D crystal structure and has dangling bonds at the GBs, which act as defects that cause deep traps or nonradiative recombination centers. (**B**) Cs_5_Cu_3_Cl_6_I_2_ consists of 1D [Cu_3_Cl_6_I_2_]*_n_*^5*n*−^ chains separated by Cs^+^ cations. The available terminations of (100) and (010) planes require no breakage of coordination bonds, which introduce no defects at the GBs, leading to a negligible nonradiative recombination at the GBs and hence short afterglow. (**C**) Radar chart of the desired properties for scintillators. A large radius is desired for an ideal scintillator. QDs, quantum dots. (**D**) Charge density plots of valence band maximum (VBM) and conduction band minimum (CBM) states for Cs_5_Cu_3_Cl_6_I_2_. The localization of electrons and holes are marked with dashed circles. (**E**) Density of states (DOS) of the bulk Cs_5_Cu_3_Cl_6_I_2_ and the planes parallel to the [001] direction, where no additional defect states are introduced within the bandgap. *E* and *E*_F_ are the energy and Fermi energy level, respectively. a.u., arbitrary units.

**Fig. 2.**
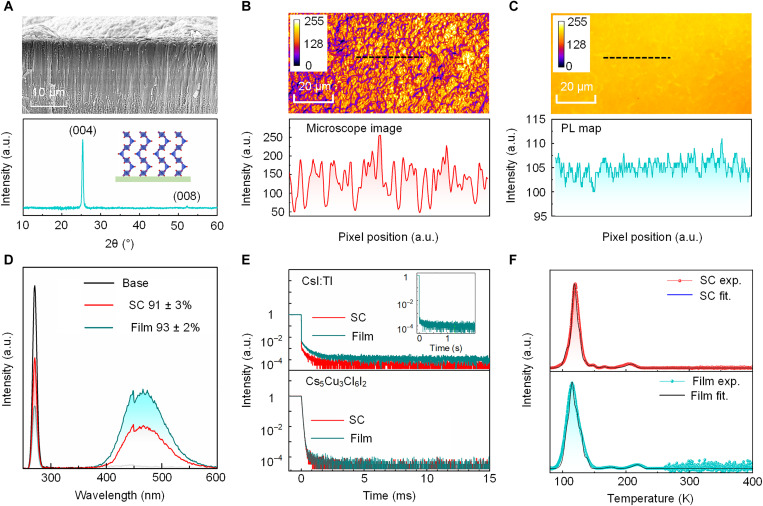
Structural and luminescence characteristics of the well-oriented Cs_5_Cu_3_Cl_6_I_2_ film. (**A**) XRD pattern (bottom) and cross-sectional SEM image of the columnar Cs_5_Cu_3_Cl_6_I_2_ film (top). (**B** and **C**) Optical microscope image (B) and photoluminescence (PL) mapping (C) of Cs_5_Cu_3_Cl_6_I_2_ film in the same position. The scanning area is 50 μm by 100 μm. Curves plot below are intensity change along the black dashed lines drawn in the same position of the microscope image and PL map. (**D**) PL quantum yield (PLQY) of Cs_5_Cu_3_Cl_6_I_2_ single crystal (SC) and film. (**E**) Time-dependent PL intensity of Cs_5_Cu_3_Cl_6_I_2_ and CsI:Tl in the state of single crystal and film. The excitation source is 292-nm laser diode. Inset is the PL residual signal of CsI:Tl film at longer time scale. (**F**) Thermally stimulated luminescence (TSL) glow curve of Cs_5_Cu_3_Cl_6_I_2_ single crystal (top) and film (bottom) with fitting by solid line (see Eq. 1 in Materials and Methods).

One of the major reasons that contributed to the long afterglow is the presence of defects at grain boundaries (GBs). The widely explored and commercially available scintillators (such as CsI:Tl, LYSO:Ce, and LaBr_3_:Ce) exhibit a three-dimensional (3D) crystal structure (*13*–*15*), that is, they are bound by covalent and/or coordination bonds in all three spatial dimensions. When processed into scintillation films, there are abundant GBs in this submillimeter-thick film. Even with a careful posttreatment, a large amount of dangling bonds inevitably present at GBs, act as nonradiative recombination centers or afterglow traps, and deteriorate device performance (Fig. 1A). For example, for CsI:Tl polycrystalline films, the light output and the afterglow characteristics are both inferior to CsI:Tl single crystals even when the best available passivation or codopants are used, largely due to the remained abundant defects at GBs (Fig. 2E and figs. S9 to S11) (*16*, *17*).

We propose a strategy using 1D crystal structure scintillation materials to address this inherent difficulty. For this 1D scintillator, atoms are bonded via strong covalent bonds into 1D chains, and these chains are then stacked together through weak van der Waals force or inert cations that do not contribute to the valence band maximum (VBM) or conduction band minimum (CBM). As a result, carriers are mostly confined within these 1D chains, in analogy to 2D materials such as graphene or MoS_2_ where carriers largely reside inside the 2D sheet (*18*). When processed into films, these 1D scintillators are prone to grow into columnar structures thermodynamically driven by the faster growth rate along the chains. Crucially, these films preserve chain integrity at GBs, eliminating detrimental dangling bonds and reducing nonradiative recombination losses and afterglow. We call these benign GBs, which are very beneficial for scintillator film for high-resolution and fast imaging (Fig. 1B). Recent examples, CsCu_2_I_3_ and A_2_CuX_3_ (A = Rb and K; X = Cl and Br), have shown potential but suffer from low luminescence efficiency [photoluminescence (PL) quantum yield (PLQY): 15.7% of CsCu_2_I_3_ and 27% of Rb_2_AgBr_3_], and the challenges in columnar film fabrication due to incongruent melting nature and the luminescence of their GBs are not studied yet (*19*–*21*). The potential of 1D materials for high-resolution, rapid scintillation imaging remains untapped.

Here, we prove the benign GBs of 1D scintillators and report the 1D Cs_5_Cu_3_Cl_6_I_2_ films for high-resolution and fast x-ray imaging. Cs_5_Cu_3_Cl_6_I_2_ consists of covalent bonded 1D [Cu_3_Cl_6_I_2_]*_n_*^5*n*−^ chains that are separated by Cs^+^ cations that have no contribution to the band edge. Moreover, Cs_5_Cu_3_Cl_6_I_2_ films bear the key features that ensure its successful scintillation application. First, Cs_5_Cu_3_Cl_6_I_2_ enjoys high stability against humidity and continuous radiation, and all the contained elements are cheap, nontoxic, and nonradioactive (*22*). The congruent melting property promises convenient fabrication via the scalable physical vapor deposition method (*23*). Combining with its 1D crystal structure, it is facile to achieve columnar structured film and benign GBs, thus allowing for high spatial resolution and low afterglow. The physical and scintillation properties of 1D Cs_5_Cu_3_Cl_6_I_2_ films are comprehensively compared with conventional and some emerging scintillators in the radar chart (Fig. 1C and table S1), outperforming most of scintillators.

## RESULTS

We began by synthesizing Cs_5_Cu_3_Cl_6_I_2_ single crystals and thick films. Previous study has reported the existence of Cs_5_Cu_3_Cl_6_I_2_ phase and its PL characteristics by studying the powder state (*24*, *25*). However, the single crystals and thick films have not been obtained, which are necessary for assessing their x-ray detection performance. Cs_5_Cu_3_Cl_6_I_2_ single crystal was obtained by the vertical Bridgman method. This compound has a congruent melting nature, as evidenced by the single endothermic peak at 339°C during a heating process (fig. S1). This property enables an easy control of the melt growth of single crystal and also the film fabrication via physical vapor deposition method. The as-polished Cs_5_Cu_3_Cl_6_I_2_ crystal slabs are shown in the inset of fig. S2. The crystals are transparent, colorless, and inclusion-free. The optical bandgap was estimated as 3.74 eV (fig. S3). Under 256-nm ultraviolet light excitation, the crystals emit bright cyan light. The x-ray diffraction (XRD) pattern and elemental mapping confirm the pure phase of Cs_5_Cu_3_Cl_6_I_2_ (fig. S4).

As shown in Fig. 1B, fig. S5, and table S2, Cs_5_Cu_3_Cl_6_I_2_ crystallizes into the orthorhombic crystal structure with the Cmcm space group, and the lattice parameters are *a* = 16.9110 Å, *b* = 9.1470 Å, and *c* = 14.0570 Å. From the view of coordination chemistry, Cu^+^ has an electronic configuration of [Ar]3d^10^, and the outer shell takes sp^3^ hybridization orbitals, while four halogen ions (Cl^−^ and I^−^) donate their lone pair electrons to coordinate with Cu^+^ to form a tetrahedron [CuCl_2_I_2_] unit. The role of Cs^+^ is to balance the charge and separate the 1D zigzag [Cu_3_Cl_6_I_2_]*_n_*^5*n*−^ chain units. According to the calculated electronic band structures and density of states, the VBM consists mainly of the localized Cu 3d orbitals and partial halogen p orbitals, rendering the valence band edge rather flat with large effective mass of holes (fig. S6). The CBM is mainly contributed from Cu 4s orbitals with some components from halogen p orbitals, and the single I^−^ ion bridging between [CuCl_2_I]_2_^4−^ and [CuCl_2_I_2_]^3−^ units further contributes to the localization of Cu 4s electron cloud in these units. Thus, although the crystal structure is 1D, the electronic dimensionality is quasi-zero, and carriers are strongly confined (Fig. 1D), which explains a large energy barrier for the exciton emission quenching (1087 meV; fig. S15C) and a high PL efficiency (PLQY = 91 ± 3% for our synthesized Cs_5_Cu_3_Cl_6_I_2_ single crystals).

For scintillator film, the columnar wave-guiding structure is well known as one of the most effective methods to reduce the light scattering, minimize the pixel cross-talk, and enable a high spatial resolution. However, it is a great challenge to obtain columnar structured films with a high aspect ratio due to the difficulty of suppressing isotropic nucleation and crystal growth. Now, only CsI:Tl film could be reproducibly processed into columnar structure. Here, the congruent melting point of Cs_5_Cu_3_Cl_6_I_2_ indicates a good phase stability in the melting state, and the 1D crystal structure makes the growth along the *c* direction thermodynamically favorable. We chose our previously established close-space sublimation (CSS) method to fabricate columnar structured films (*26*). The Cs_5_Cu_3_Cl_6_I_2_ powder was heated to 600°C in 60 s and deposited onto a 6 cm–by–6 cm quartz substrate. The XRD pattern (Fig. 2A) shows the excellent orientation along the [004] direction of the Cs_5_Cu_3_Cl_6_I_2_ film, where the 1D zigzag [Cu_3_Cl_6_I_2_]*_n_*^5*n*−^ chains are perpendicular to the substrate (Fig. 2A, inset). The scanning electron microscopy (SEM) image shows the grain size of 2 to 5 μm in diameter (Fig. 2A and fig. S7), which was smaller than commercial CsI:Tl film (*7*).

Furthermore, we use the first-principles calculations to study the atomic and electronic structure of Cs_5_Cu_3_Cl_6_I_2_ crystallographic planes. The available terminations of (100) and (010) planes require no breaking of Cu─X coordination bonds, and the plane energy is expected to be lower than in the case of the planes with broken coordination bonds. The calculation results reveal that the plane energies for (100), (010), and (001) planes are 0.27, 0.34, and 0.40 J m^−2^, respectively, consistent with the expectations. This indicates that the most abundant planes are the (100) and (010) faces, due to their lower formation energy and no breakage of coordination bonds, in good agreement with XRD results. For all the planes considered, no substantial plane reconstruction was observed in the calculations (fig. S8). Following electronic structure calculation shows that no deep states are present inside the bandgap for the (100) and (010) planes (Fig. 1E and fig. S8). Thus, the GBs will be terminated with the intrinsically benign planes, and the traps causing the afterglow and nonradiative recombination losses could be eliminated.

To support these theoretical predictions, we experimentally compare the luminescence properties of the GBs and grain interiors. The 2D topography optical microscopy image and the corresponding PL mapping of the Cs_5_Cu_3_Cl_6_I_2_ films (Fig. 2, B and C) show that there is no correlation between GBs (identifiable by notable changes in topography spatial map) and the PL intensity variations. In an illustrative line crossing the GBs, the luminescence intensity of the grain interiors is 105 ± 1.28 and that of GBs is 104 ± 1.36. The average luminescence intensity difference is extremely small (1.40), which is comparable to the noise fluctuation. In comparison, for CsI:Tl scintillator film, along the illustrative line across three CsI:Tl grains with identical height, the PL intensity severely reduces from 248 ± 3.28 at the grain interiors to 233 ± 3.73 at GBs (fig. S9), which is caused by the harmful dangling bonds at GBs.

Moreover, we measured the PLQYs of single crystal and polycrystalline films of Cs_5_Cu_3_Cl_6_I_2_ and CsI:Tl. As shown in Fig. 2D, the PLQY of Cs_5_Cu_3_Cl_6_I_2_ single crystal is 91 ± 3%, and the Cs_5_Cu_3_Cl_6_I_2_ film exhibits similar PLQY of 93 ± 2%. By contrast, the PLQY of CsI:Tl single crystal is 98 ± 1%, while PLQY of CsI:Tl film is markedly reduced to 85 ± 1% (fig. S10), which is attributed to the light quenching by numerous surface states at the GBs (*27*, *28*). As shown in Fig. 2E, there is also no obvious difference in PL decay between Cs_5_Cu_3_Cl_6_I_2_ single crystal and film, where the decay time constants can be well fitted by a single exponential function as 39.0 and 38.1 μs, respectively. The PL residual signal of Cs_5_Cu_3_Cl_6_I_2_ single crystal and film under band-to-band excitation is 0.01% at 0.70 ms and 0.01% at 0.69 ms, respectively. Such a fast decrease of PL signal with the same time profile in single crystal and film indicates that there is no notable effect of additional trapping centers at GBs. In sharp contrast, for CsI:Tl, the film clearly produces a longer decay tail (time constant of 540.7 ms; fig. S11) than the single crystal. The PL residual signal of CsI:Tl single crystal is 0.01% at 1.08 ms, while in the film is 0.01% at 1.25 s, i.e., much more intense trapping effects are evident in the latter (Fig. 2E, inset).

Thermally stimulated luminescence (TSL) is a powerful technique to probe the trapping and detrapping dynamics of carriers from defects (*29*). Near–room temperature TSL directly reflects the deep traps, which are the culprit for afterglow effect. From the TSL glow curves as shown in Fig. 2F, the Cs_5_Cu_3_Cl_6_I_2_ single crystal and film have almost the same TSL peak position and peak shape. A general order kinetic function was used to fit the glow curves and evaluate the trap parameters (*30*), i.e., the energetic depth of the traps, the attempt-to-escape frequency factor, and the detrapping time. The trap depth and detrapping time of the defect state in the single crystal and film are almost the same (table S3), which suggest that there is no additional defect state at GBs in the film. Notably, there is only one trap state with detrapping time in the millisecond level, which is 0.57 eV (3.10 ms) and 0.60 eV (5.26 ms) for the single crystal and film, respectively. Moreover, the glow intensity of Cs_5_Cu_3_Cl_6_I_2_ single crystal in 260 to 500 K is about two orders magnitude lower than that of CsI:Tl single crystal measured at identical conditions (fig. S12), indicating a much lower deep trap density of Cs_5_Cu_3_Cl_6_I_2_.

Both theoretical simulation and experimental results revealed that, for our [001]-oriented Cs_5_Cu_3_Cl_6_I_2_ films, a negligible difference was observed between films and single crystals, indicating that grain interiors and GBs are nearly indistinguishable and the GBs are intrinsically benign. We admit that some point defects (for example, I vacancies) or contamination could still exist at GBs, but defects from dangling bonds are largely eliminated, minimizing afterglow and nonradiative recombination losses. This is in sharp contrast with 3D scintillators such as CsI:Tl, where a huge difference was observed between its single crystal and film due to the abundant dangling bonds and consequently amounts of defects states at GBs.

**Fig. 3.**
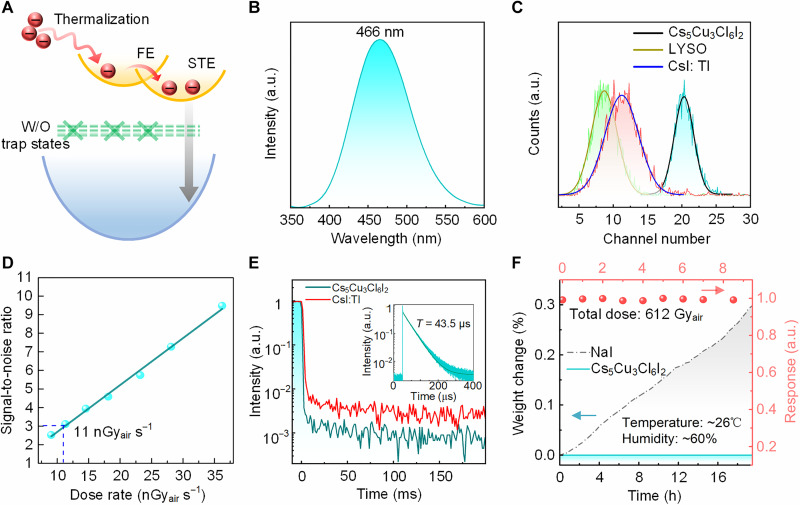
The scintillation performance of Cs_5_Cu_3_Cl_6_I_2_. (**A**) Scintillation process of Cs_5_Cu_3_Cl_6_I_2_ under x-ray excitation. FE, free exciton; STE: self-trapped exciton. W/O, without. (**B**) Radioluminescence (RL) spectrum of Cs_5_Cu_3_Cl_6_I_2_. The peak position is at 466 nm with a full width at half maximum (FWHM) of 86 nm. (**C**) Pulse height spectrum of LYSO:Ce, CsI:Tl, and Cs_5_Cu_3_Cl_6_I_2_ for ^241^Am (59.5 keV). The scintillation yield was calculated by correcting the wavelength-dependent detection efficiency of PMT for these three scintillators. (**D**) The linear response curve of Cs_5_Cu_3_Cl_6_I_2_ film toward x-ray and the detection limit was determined at the signal-to-noise ratio (SNR) as 3. (**E**) Afterglow profile of Cs_5_Cu_3_Cl_6_I_2_ and CsI:Tl film after x-ray cutoff. Inset is the scintillation decay profile of Cs_5_Cu_3_Cl_6_I_2_ under the pulsed x-ray excitation. (**F**) Stability of Cs_5_Cu_3_Cl_6_I_2_ single crystal toward humidity and continuous radiations. NaI:Tl single crystal with the same size was used as the reference.

Now, we investigate the scintillation properties of Cs_5_Cu_3_Cl_6_I_2_. Cs_5_Cu_3_Cl_6_I_2_ contains high-*Z* elements of Cs and I and has an average atomic number of 49, which guarantees high x-ray attenuation efficiency (table S4). According to the photon cross-sectional database, the attenuation coefficient of Cs_5_Cu_3_Cl_6_I_2_ is comparable to that of CsI:Tl (fig. S13). The scintillation process of Cs_5_Cu_3_Cl_6_I_2_ under x-ray excitation is described in Fig. 3A. High-energy electrons induced by interaction of x-ray with atoms in the Cs_5_Cu_3_Cl_6_I_2_ produce an avalanche of secondary electrons that are thermalized and lastly coupled with surrounding crystal lattice to form self-trapped excitons (STEs) and emit photons. The nature of STE emission is judged from the broad emission spectrum, large Stokes shift (fig. S14), and the soft lattice of Cs_5_Cu_3_Cl_6_I_2_. There are two emission centers (480 nm, STE1; and 590 nm, STE2) of Cs_5_Cu_3_Cl_6_I_2_ under 77 K, which can be correlated with the deformation of different Cu─Cu bonds in the zigzag chains. The Huang-Rhys factors (*S*) of two emission centers are derived from the full width at half maximum (FWHM) of PL as 87 and 48 (fig. S15). The large *S* values indicate the existence of strong electron-phonon coupling, which also support the STE emission nature. As shown in Fig. 3B, the x-ray excited radioluminescence (RL) spectrum has a single emission peak at 466 nm with an FWHM of 86 nm, which is identical to its PL spectrum (fig. S14).

The scintillation yield of Cs_5_Cu_3_Cl_6_I_2_ is calculated by comparing its ^241^Am (59.5 keV) excited pulse height spectrum with that of LYSO:Ce and CsI:Tl references. The scintillators were fixed on a photomultiplier tube (PMT) with optical coupling glue. The scintillation pulses were recorded by an oscilloscope and related pulse height spectra are shown in Fig. 3C. The scintillation yield is proportional to the channel number of the full energy peak centroid and was further corrected by considering the emission-weighted quantum efficiency of PMT for these three different scintillators. The scintillation yield of Cs_5_Cu_3_Cl_6_I_2_ is 2.8 times of benchmarking LYSO:Ce (24,000 photons/MeV) and 1.2 times of CsI:Tl sample (54,000 photons/MeV). Thus, the scintillation yield of Cs_5_Cu_3_Cl_6_I_2_ is estimated within 64,800 to 67,200 photons/MeV. High scintillation yield allows scintillator detector to maintain high signal-to-noise ratios (SNRs) under low-dose irradiation. The detection limit of Cs_5_Cu_3_Cl_6_I_2_ achieves 11 nanogray equivalent air (nGy_air_) s^−1^ (Fig. 3D), which is one of the lowest among all kinds of scintillator and comparable to semiconductor x-ray detectors (CsPbBr_3_ quantum dots, 13 nGy_air_ s^−1^; Cs_0.15_FA_0.85_PbI_3_ semiconductor detector, 13.8 nGy_air_ s^−1^; and CsPbBrI_2_, 1 nGy_air_ s^−1^) (*10*, *31*, *32*), and is only 1/500 of the dose rate used in traditional x-ray medical diagnostics (5.5 μGy_air_ s^−1^) (*33*).

We recorded the scintillation afterglow of Cs_5_Cu_3_Cl_6_I_2_ and CsI:Tl film under x-ray excitation (Fig. 3E). After x-ray cutoff, the afterglow signal of Cs_5_Cu_3_Cl_6_I_2_ attenuates by nearly three orders of magnitude within 10 ms (0.1% at 10 ms), which is lower than CsI:Tl (0.4% at 10 ms) and comparable to Gd_2_O_2_S:Tb (table S1). In longer time scale (100 s), it attenuates nearly four orders of magnitude and is over one order of magnitude lower than that of CsI:Tl (fig. S17). As shown in the fig. S12, the CsI:Tl has high intensity glow peaks in 260 to 500 K, which refer to the deep trap states and result in unacceptable afterglow within seconds to minutes. We also measured the RL decay profile of Cs_5_Cu_3_Cl_6_I_2_ film under x-ray excitation (Fig. 3E, inset). The RL decay can be well fitted by a single exponential function with a time constant of 43.5 μs, similar to the PL decay lifetime of 37.4 μs (fig. S16). The similar lifetime and the same RL and PL spectrum indicate that no additional recombination pathway contributes to the scintillation emission mechanism.

We also study the stability of Cs_5_Cu_3_Cl_6_I_2_ crystal toward moisture and continuous irradiations (Fig. 3F). There is no weight change after 12 hours of exposure under a relative humidity 60%. In sharp contrast, the weight of NaI:Tl rapidly increases by 0.18% under the same condition. For radiation stability, the single crystal was exposed under x-rays in ambient atmosphere (25°C, 70% humidity) without any encapsulation, and its light output was recorded. As shown in the red dots of Fig. 3F, 8.5 hours of x-ray radiation with an accumulated total dose of 612 Gy_air_ did not deteriorate the light output of Cs_5_Cu_3_Cl_6_I_2_.

**Fig. 4.**
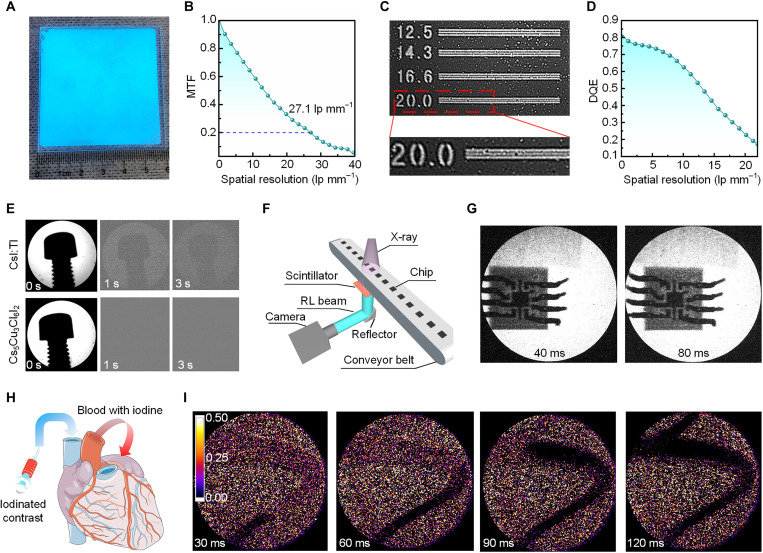
High-resolution and high-speed x-ray imaging based on Cs_5_Cu_3_Cl_6_I_2_ film. (**A**) Photograph of Cs_5_Cu_3_Cl_6_I_2_ film under ultraviolet excitation. The area of the film is 6 cm by 6 cm. (**B**) MTF of the x-ray imager. (**C**) X-ray image of a partial region (from 12.5 to 20 lp mm^−1^) of the standard x-ray resolution pattern plate. Note that the “white dots” in the image are caused by the camera response to the scattered x-rays. (**D**) Detective quantum efficiency (DQE) of the x-ray imager. (**E**) Dynamic imaging of a screw after 10 s of x-ray excitation using CsI:Tl and Cs_5_Cu_3_Cl_6_I_2_ film. The Cs_5_Cu_3_Cl_6_I_2_ film shows no residual image, while the image in CsI:Tl film persists even after 3 s. (**F**) The schematic diagram of chips manufacturing pipelining inspection. (**G**) X-ray imaging of a moving chip. (**H**) The schematic diagram of angiography. (**I**) The demonstration of angiography by using Cs_5_Cu_3_Cl_6_I_2_ film. The iodinated contrast media flow in the hose can be clearly seen.

Last, we studied the x-ray imaging performance of Cs_5_Cu_3_Cl_6_I_2_ film. We note that, through our CSS process, the Cs_5_Cu_3_Cl_6_I_2_ film could be easily and reproducibly deposited onto various substrates, including quartz (Fig. 4A), flexible polyimide (fig. S18), or thin-film transistor (TFT) panels due to its congruent melting property. According to the slanted-edge method, a high spatial resolution of 27.1 line pairs per millimeter (lp mm^−1^) at a modulation transfer function (MTF) of 0.2 is obtained for the Cs_5_Cu_3_Cl_6_I_2_ film (Fig. 4B). This value far exceeds commercial CsI:Tl (3 to 5 lp mm^−1^) and GOS:Tb (1.7 lp mm^−1^) and also exceed that of the recently reported record spatial resolution of perovskite (16.8 lp mm^−1^) and organic scintillators (18 lp mm^−1^) (*10*, *34*–*38*). The high spatial resolution can be attributed to the smaller columnar crystals, which reduce optical cross-talk (*39*). Furthermore, we took photos of a standard x-ray resolution pattern plate (QUART Type 74, 5.0 to 20 lp mm^−1^) and gold wires with 15 μm diameter (Fig. 4C and fig. S19). The results demonstrate that the line pair notches at 20 lp mm^−1^ and the gold wires are clearly distinguishable, echoing the results by the slanted-edge method.

For imaging application, the detective quantum efficiency (DQE) is an important metric related to the image quality, which refers to the efficiency of an x-ray detector in converting x-ray photons into imaging signal. As shown in Fig. 4D, the DQE(0) of Cs_5_Cu_3_Cl_6_I_2_ film is about 80%, which is larger than that of commercial CsI:Tl-based complementary metal-oxide semiconductor (CMOS) FPD (60 to 75%) and GOS:Tb-based FPD (35%) (*38*, *40*). In addition, the DQE of the Cs_5_Cu_3_Cl_6_I_2_ film decreases slowly with spatial resolution and remains above 20% even at 23 lp mm^−1^. The high DQE can primarily be attributed to the exceptional spatial resolution of the Cs_5_Cu_3_Cl_6_I_2_. High DQE ensures that the Cs_5_Cu_3_Cl_6_I_2_ film maintains a good contrast for small objects imaging and enables its competitive application in x-ray FPD. We thus demonstrated the high-quality x-ray images of a print circuit board and a spring (fig. S20). The outline of the invisible connection cable and spring were clearly presented. Our Cs_5_Cu_3_Cl_6_I_2_ films have minimized recombination loss at GBs and high scintillation yield, enabling large SNR and hence large DQE and sensitive detection; they also have columnar structure with a large refractive index (1.77 at 410 nm; fig. S21), resulting in a negligible cross-talk and hence a high spatial resolution. It is also worth mentioning that the Cs_5_Cu_3_Cl_6_I_2_ film also exhibits high stability. After being exposed to air for 3 months, there was no noticeable change observed in its PLQY (fig. S22A). Furthermore, the light output and imaging ability remain excellent even after continuous irradiation (fig. S22, B and C).

As stated before, the Cs_5_Cu_3_Cl_6_I_2_ scintillator enjoys a low afterglow. We used a camera to acquire residual images of a screw after 10 s of x-ray lasting exposition. As shown in Fig. 4E, the Cs_5_Cu_3_Cl_6_I_2_ film shows virtually no residual image in any of the frames, whereas the commercial CsI:Tl film shows a clearly residual image even after 3 s.

Last, we showcase our Cs_5_Cu_3_Cl_6_I_2_ film for high-resolution and fast x-ray imaging, a long-sought goal for x-ray FPD. As shown in Fig. 4F, the chips (fig. S23A) were placed on a conveyor belt and x-ray was irradiated continuously. The camera recorded the image every 40 ms. As the chip moved, the chip’s internal structure could be seen very clearly and without any residual shadow (Fig. 4G and movie S1). Moreover, we also simulated the applications of Cs_5_Cu_3_Cl_6_I_2_ film in angiography, which is a medical imaging technique used to visualize the blood vessels. During angiography, a radio-opaque contrast agent needs to be injected into the body, such as iodinated contrast media, flowing through the heart to the whole body (Fig. 4H). Here, we simulate the process by injecting the iodinated contrast media into a hose (fig. S23, B to D). The flow position of the contrast media in the hose can be clearly observed with a frame interval of 30 ms (Fig. 4I and movie S2). This speed outperforms traditional angiographic imaging in clinical settings and substantially reduces the stroboscopic effects (*41*). The detection of blood flow rate by our Cs_5_Cu_3_Cl_6_I_2_ scintillator is remarkable as this is indispensable for the diagnosis and monitoring of cardiovascular disease.

## DISCUSSION

In summary, we successfully developed a class of x-ray imagers based on 1D scintillator film and demonstrated its unprecedented capability for high-resolution and fast x-ray imaging. Compared to conventional 3D scintillators that suffer from defects at GBs, 1D scintillators are prone to grow into columnar films with inert planes exposed and, thereafter, enjoy benign GBs and enable minimized recombination loss and suppressed afterglow. Our rationally selected 1D scintillator Cs_5_Cu_3_Cl_6_I_2_ exhibits the long-sought combination of high scintillation yield (1.2 times of CsI:Tl), low afterglow (0.1% at 10 ms), columnar growth capability, low toxicity, and good stability. As a result, our Cs_5_Cu_3_Cl_6_I_2_ x-ray imager achieved a high spatial resolution of 27.1 lp mm^−1^ and a DQE(0) of 80%, exceeding nearly all reported scintillators. Moreover, the concept of 1D scintillator can be further exploited for other high-speed and high–spatial resolution x-ray imaging applications.

## MATERIALS AND METHODS

### Materials

CuCl (99.999%) was purchased Alfa Aesar. CuI (99.995%) was purchased from Acros Organics. CsCl (99.999%) and CsI (99.999%) were purchased from Advanced Election Technology Co. Ltd. All reagents were used as received.

### Cs_5_Cu_3_Cl_6_I_2_ single crystal

High-purity CsCl, CuCl, and CuI were used as received. The raw materials were mixed and loaded in a 7-mm-diameter quartz ampoule in an Ar glovebox according to the stoichiometric of 5:1:2 for CsCl:CuCl:CuI. The ampoule was sealed under a vacuum of 10 Pa and placed into the vertical Bridgman growth furnace. The growth temperature was about 330°C. The temperature gradient of about 20°C cm^−1^ and the growth rate of 0.5 mm h^−1^ were used. After the crystal growth was completed, the furnace temperature was cooled to room temperature in 24 hours.

### Cs_5_Cu_3_Cl_6_I_2_ film

The Cs_5_Cu_3_Cl_6_I_2_ film was deposited on a quartz glass or polyimide substrate by a CSS system (Qihui Vacuum Technology Co. Ltd. Shenyang, China). The graphite boat source was filled with 0.8 g of Cs_5_Cu_3_Cl_6_I_2_ powder, which was obtained by grinding the single crystals. The source and the substrate were placed within a quartz tube, and the whole system was kept under a vacuum below 2 × 10^−3^ Pa. The source was heated to 600°C within 60 s via infrared radiation. The sublimated Cs_5_Cu_3_Cl_6_I_2_ species condensed on the substrate to form the Cs_5_Cu_3_Cl_6_I_2_ film with a thickness of 30 μm.

### Theoretical calculation

The density functional theory calculations were performed by the Vienna Ab initio Simulation Package (*42*). The Perdew-Burke-Ernzerhof generalized gradient approximation was used for the exchange-correlation functional (*43*). Projector-augmented wave potentials were used to describe the interactions between ion cores and valence electrons (*44*). Plane waves with cutoff energy of 22.5 Ry were taken as the basis set. For the electronic structure calculation of bulk materials, we used experimentally determined crystal structure. The first Brillouin zone was sampled by a Γ-centered 3 × 4 × 3 *k*-point grid. To calculate the plane energy, plane structures based on 2 × 1 × 1, 1 × 2 × 1, and 1 × 1 × 2 supercells were constructed for (100), (010), and (001) planes, respectively. A vacuum layer of at least 10 Å was used. The atoms were relaxed until the residual forces on all atoms were smaller than 0.01 eV. Then, the plane energy was calculated on the basis of a Γ-centered *k*-point grid of 4 × 2 × 1.

### Material characterization

Single-crystal x-ray diffraction was collected with a graphite-monochromatized Mo *K*α radiation (λ = 0.71073 Å) at room temperature on a Bruker-Lynxeye diffractometer. The data reduction and multiscan absorption correction were all performed using the CrysAlisPro software supplied by the manufacturer. Ab initio structure solutions were performed by the Direct Methods algorithm using ShelXS program. All structures were refined by full-matrix least squares using ShelXL program. The morphology of the cross section and EDS mapping was studied by SEM (GeminiSEM 300, Carl Zeiss) with a 10-kV accelerating voltage. The differential scanning calorimetry was conducted by using a Netzsch STA449F3 instrument. About 50 mg of the single-crystal sample was heated to 400°C in Al_2_O_3_ crucible at a rate of 5°C/min under the condition of ultrahigh purity argon atmosphere.

The PLE and emission (PL) spectra, emission and excitation contour mapping, and RL spectrum and x-ray–induced afterglow spectra at room temperature were measured with a Horiba FluoroMax+ spectrofluorometer. A Xe steady-stay lamp was used as the excitation source for PL measurements. For RL measurements, an x-ray tube (50 kV, 500 mA, KYW900A) with W anode was used as the excitation source. An integrating sphere was equipped to collect the emissions, which was fed through an optical fiber to the receiving end of the spectrofluorometer with dual scanning monochromators. The PL decay spectrum was recorded by a QuantaMaster 8000 (HORIBA Scientific, Canada) with an excitation wavelength at 290 nm. The PLQY was determined by an absolute quantum yield measurement system attached with an integrating sphere and excited by 271-nm wavelength (Hamamatsu Quantaurus-QY). The PL mapping was measured by a fluorescence microscope (BX53, Olympus, Japan).

TSL was measured by ROSB TL 3DS thermoluminescence spectrometer. The CsI:Tl and Cs_5_Cu_3_Cl_6_I_2_ single crystal has the same size of 4 mm by 4 mm by 2 mm. The samples were irradiated by x-ray (50 kV, 500 mA) for 10 min at 80 K and 30 min of waiting and then heated at a heating rate of 0.1 K s^−1^. The curves were fitted by following equation

$$I(T)={sn}_{0}e^{-\frac{E}{k_{B}T}}\times {\left\{\frac{(l-1)s}{\beta }\times T\times e^{-\frac{E}{k_{B}T}}\times \left[\frac{k_{B}T}{E}-2{\left(\frac{k_{B}T}{E}\right)}^{2}+6{\left(\frac{k_{B}T}{E}\right)}^{3}\right]+1\right\}}^{l/(l-1)}$$ (1)

where *E* is the trap depth, *s* is the frequency factor, *n*_0_ is the initial concentration of trapped charges, *l* is the kinetic order, *k*_B_ is the Boltzmann constant, and β is the heating rate.

The PLE and emission (PL) spectra at 77 K and the temperature dependence spectra were measured by Xe lamp excitation via Edinburgh Instruments FLS 980 spectrometer with a temperature controller for liquid nitrogen (77 to 500 K) cryostat. The activation energy (*E*_a_) was estimated by fitting the integrated PL intensity under different temperatures with the following equation

$$\frac{I(T)}{I_{0}}=\frac{1}{1+\frac{{\text{Γ}}_{0}}{{\text{Γ}}_{\nu }}\exp\left(-\frac{E_{a}}{k_{B}T}\right)}$$ (2)

where *I*_0_ is the integral intensity at 0 K, Γ_ν_ is the radiative decay rate, Γ_0_ is the attempt rate of thermal quenching, and *k*_B_ is the Boltzmann constant. The Huang-Rhys factor (*S*) was obtained by nonlinear fitting the temperature dependent FWHM with the equation (*45*)

$$\mathrm{FWHM}=2.36\sqrt{S}\hbar \omega_{\mathrm{phonons}}\sqrt{coth\frac{\hbar \omega_{\mathrm{phonons}}}{2k_{B}T}}$$ (3)

where *ℏ*ω_phonons_ is the phonon frequency.

The refractive index was measured by a spectroscopic ellipsometry (SE-VM-L, Wuhan Eoptics Technology Co. Ltd.) with measurement wavelength of 380 to 1000 nm. The light spot diameter was 200 μm. A Cs_5_Cu_3_Cl_6_I_2_ single crystal with 8-mm diameter and 2-mm thickness was used in the measurement. The crystal was polished and guaranteed that the top and bottom surfaces were parallel to each other.

### Scintillation characterization

The scintillation yield was calculated by comparison of the ^241^Am pulse height spectra. The Cs_5_Cu_3_Cl_6_I_2_ crystal was a cylinder with a diameter of 7 mm and a thickness of 3 mm. The CsI:Tl crystal (purchased from Scionix) was a cube with a size of 3 mm by 3 mm by 5 mm, and the size of LYSO crystal was 3 mm by 3 mm by 10 mm. The density of Cs_5_Cu_3_Cl_6_I_2_, CsI:Tl, and LYSO was 3.8, 4.5, and 7.1 g cm^−3^, respectively. The crystal was fixed on a PMT (R2059, Hamamatsu) by optical coupling glue. A high voltage power supply (556, Ortec) was used to supply voltage to the PMT. An oscilloscope (MSO54B 5-BW-2000, Tektronix) was used to read the output signal of the PMT. The ^241^Am with radioactivity of 2.85 × 10^8^ Bq irradiated the crystal through a Be window from a distance of 1 cm. The PMT and ^241^Am were placed in shielded boxes and completely protected from light. A total of 5000 pulses were collected by the oscilloscope and processed as pulse height spectra and fitted by Gauss function. The relative scintillation yield of Cs_5_Cu_3_Cl_6_I_2_ was obtained by following equation

$$LY_{R}=\frac{C_{\mathrm{Cs}5\mathrm{Cu}3\mathrm{Cl}6I2}}{C_{\mathrm{CsI}:\mathrm{Tl}/\mathrm{LYSO}}}\cdot \frac{\phi_{\mathrm{CsI}:\frac{\mathrm{Tl}}{\mathrm{LY}\mathrm{SO}}}}{\phi_{\mathrm{Cs}5\mathrm{Cu}3\mathrm{Cl}6I2}}$$ (4)

where *LY*_R_ was the relative scintillation yield, *C_j_* (*j* = Cs_5_Cu_3_Cl_6_I_2_/CsI:Tl/LYSO) was the channel number, and ϕ*_j_* was the PMT spectral quantum efficiency (fig. S24A) correction constant. The PMT spectral quantum efficiency correction constant was calculated by

$$\phi_{j}=\int I_{j}(\lambda )S(\lambda )d\lambda /\int I_{j}(\lambda )d\lambda$$ (5)

where *I_j_*(λ) was the RL intensity of scintillator (fig. S24B) and *S*(λ) was the wavelength-dependent detection efficiency of PMT.

For the detection limit measurement, the Cs_5_Cu_3_Cl_6_I_2_ was attached to a silicon photomultiplier (JSP-TN3050-SMT), and the data were collected by an oscilloscope (DSO-S 054A, Keysight). The x-ray dose rates were adjusted by changing the current of the x-ray tube (10 to 80 mA, 50 kV; Leo, Varex Imaging) and increasing the Cu filter (0 to 2 mm). The dose rates were calibrated with an ion chamber (10X6-180, Radcal) and a solid-state sensor (DDX6-WL). We measured the scintillator response under dose rates of 9.05, 11.31, 14.56, 18.11, 23.21, 28.11, and 36.22 nGy_air_ s^−1^. The detection limit was derived when the SNR of the response equaled to 3. As shown in fig. S25, the “signal” was calculated by subtracting the average value of the dark voltage from the photovoltage. The “noise” was the SD of the photovoltage.

The scintillation decay was obtained by using x-ray tube (70 kV, 10 ns of pulse width) with W anode as excitation source, an optical fiber to transmit the light to PMT (2 ns of rise time), and a digital phosphor oscilloscope (1 GHz of bandwidth) to get the curve.

The afterglow was obtained by using x-ray tube (10 kV, 50 mA, Seifert Gmbh) for excitation and collecting the spectrally unresolved emission by 5000 M model of Horiba Jobin Yvon fluorometer running in the multichannel scaling mode and equipped with TBX-04 photon counting detector (IBH-Scotland).

### X-ray imaging

We assembled an imaging system in a black lead box. The x-ray source used in the system was M237 (50 kV, Newton Scientific) with Au target. The Cs_5_Cu_3_Cl_6_I_2_ film was placed on a reflector (CCM1-G01, Thorlabs), and the scintillation light was deflected to a CMOS camera (C13440, Hamamatsu) with pixel size of 6.5 μm by 6.5 μm. For the demonstration of chip pipelining inspection, a chip was fixed to a scanning stage (PSA200-11-X, Zolix) at a step rate of 4.5 mm s^−1^. The camera was set at automatic acquisition mode. For the angiography simulation, a processing approach typical of digital subtraction angiography was applied. A “mask image” was captured before the injection of the contrast agent. This mask image was then subtracted from the subsequent image taken after contrast injection, yielding a clear angiographic image. The pixel values of scintillation photon noise and the camera’s electronic noise were calculated as 6.4 (fig. S26).

DQE was calculated with MIQuaELa software and measured following the guidance of the international standard IEC62372. The noise power spectrum (NPS; fig. S27) and MTF were measured with RQA3 beam, which was produced form a tube (Leo, Varex Imaging) voltage of 50 kV. The detector responses were measured under several different doses to obtain signal transfer property. The absorption dose of 2.5 μGy_air_ was used to measure the NPS of the detector.

## Supplementary Materials

### This PDF file includes:

Supplementary Text Figs. S1 and S27 Tables S1 to S4 Legends for movies S1 and S2 References

## References

[1] P. Russo, Ed., in *Handbook of X-ray Imaging: Physics and Technology* (CRC Press, 2017), p. 1419.

[2] K. Chrzanowski, Review of night vision metrology. Opto-Electronics Rev. 23, 149–164 (2015).

[3] R. Autrata, P. Schauer, J. Kuapil, J. Kuapil, A single crystal of YAG-new fast scintillator in SEM. J. Phys. E 11, 707–708 (1978).

[4] E. Auffray, F. Cavallari, M. Lebeau, P. Lecoq, M. Schneegans, P. Sempere-Roldan, Crystal conditioning for high-energy physics detectors. Nucl. Inst. Methods Phys. Res. A 486, 22–34 (2002).

[5] C. Dujardin, E. Auffray, E. Bourret-Courchesne, P. Dorenbos, P. Lecoq, M. Nikl, A. N. Vasil’Ev, A. Yoshikawa, R. Y. Zhu, Needs, trends, and advances in inorganic scintillators. IEEE Trans. Nucl. Sci. 65, 1977–1997 (2018).

[6] T. Martin, A. Koch, Recent developments in X-ray imaging with micrometer spatial resolution. J. Synchrotron Radiat. 13, 180–194 (2006). 16495618 10.1107/S0909049506000550

[7] V. V. Nagarkar, T. K. Gupta, S. R. Miller, Y. Klugerman, M. R. Squillante, G. Entine, Structured CsI(Tl) scintillators for X-ray imaging applications. IEEE Trans. Nucl. Sci. 45, 492–496 (1998).

[8] V. V. Nagarkar, S. C. Thacker, V. Gaysinskiy, L. E. Ovechkina, S. R. Miller, S. Cool, C. Brecher, Suppression of afterglow in microcolumnar CsI:Tl by codoping with Sm: Recent advances. IEEE Trans. Nucl. Sci. 56, 565–569 (2009). 20617107 10.1109/tns.2009.2016198 PMC2898182

[9] Y. Wu, G. Ren, F. Meng, X. Chen, D. Ding, H. Li, S. Pan, Effects of Bi^3+^ codoping on the optical and scintillation properties of CsI:Tl single crystals. Phys. Status Solidi 211, 2586–2591 (2014).

[10] Q. Chen, J. Wu, X. Ou, B. Huang, J. Almutlaq, A. A. Zhumekenov, X. Guan, S. Han, L. Liang, Z. Yi, J. Li, X. Xie, Y. Wang, Y. Li, D. Fan, D. B. L. Teh, A. H. All, O. F. Mohammed, O. M. Bakr, T. Wu, M. Bettinelli, H. Yang, W. Huang, X. Liu, All-inorganic perovskite nanocrystal scintillators. Nature 561, 88–93 (2018). 30150772 10.1038/s41586-018-0451-1

[11] C. Greskovich, S. Duclos, Ceramic scintillators. Annu. Rev. Mater. Sci. 27, 69–88 (1997).

[12] C. W. E. van Eijk, Inorganic scintillators in medical imaging detectors. Nucl. Inst. Methods Phys. Res. A 509, 17–25 (2003).

[13] G. Blasse, B. C. Grabmaier, *Luminescent Materials* (Springer, 1994).

[14] D. Chiriu, N. Faedda, A. G. Lehmann, P. C. Ricci, A. Anedda, S. Desgreniers, E. Fortin, Structural characterization of Lu_1.8_Y_0.2_SiO_5_ crystals. Phys. Rev. B - Condens. Matter Mater. Phys. 76, 054112 (2007).

[15] L. Labr, K. Kramer, M. Schulze, Three bromides of lanthanum: LaBr_2_, La_2_Br_5_, and LaBr_3_. Z. Anorg. Allg. Chem. 575, 61–70 (1989).

[16] Y. Zorenko, T. Voznyak, R. Turchak, A. Fedorov, K. Wiesniewski, M. Grinberg, Luminescent and scintillation properties of CsI:Tl films grown by the liquid phase epitaxy method. Phys. Status Solidi Appl. Mater. Sci. 207, 2344–2350 (2010).

[17] A. Fedorov, A. Lebedinsky, O. Zelenskaya, Scintillation efficiency, structure and spatial resolution of CsI(Tl) layers. Nucl. Instr. Meth. Phys. Res. A 564, 328–331 (2006).

[18] M. Chhowalla, D. Jena, H. Zhang, Two-dimensional semiconductors for transistors. Nat. Rev. Mater. 1, 16052 (2016).

[19] R. Lin, Q. Guo, Q. Zhu, Y. Zhu, W. Zheng, F. Huang, All-inorganic CsCu_2_I_3_ single crystal with high-PLQY (≍15.7%) intrinsic white-light emission via strongly localized 1D excitonic recombination. Adv. Mater. 31, 1905079 (2019). 10.1002/adma.201905079 31583772

[20] M. Zhang, X. Wang, B. Yang, J. Zhu, G. Niu, H. Wu, L. Yin, X. Du, M. Niu, Y. Ge, Q. Xie, Y. Yan, J. Tang, Metal halide scintillators with fast and self-absorption-free defect-bound excitonic radioluminescence for dynamic X-ray imaging. 31, 2007921 (2021).

[21] B. Yang, L. Yin, G. Niu, J. Yuan, K. Xue, Z. Tan, X. Miao, M. Niu, X. Du, H. Song, E. Lifshitz, J. Tang, Lead-free halide Rb_2_CuBr_3_ as sensitive X-ray scintillator. Adv. Mater. 31, e1904711 (2019). 31531905 10.1002/adma.201904711

[22] M. Baskaran, *Handbook of Environmental Isotope Geochemistry* (Springer, 2012).

[23] S. O. Ferreira, *Advanced Topics on Crystal Growth* (InTech, 2013).

[24] J. Li, T. Inoshita, T. Ying, A. Ooishi, J. Kim, H. Hosono, A highly efficient and stable blue-emitting Cs_5_ Cu_3_ Cl_6_ I_2_ with a 1D chain structure. Adv. Mater. 32, 2002945 (2020). 10.1002/adma.202002945 32761681

[25] X. Niu, J. Xiao, B. Lou, Z. Yan, Q. Zhou, T. Lin, C. Ma, X. Han, Highly efficient blue emissive copper halide Cs_5_Cu_3_Cl_6_I_2_ scintillators for X-ray detection and imaging. Ceram. Int. 48, 30788–30796 (2022).

[26] M. Zhang, J. Zhu, B. Yang, G. Niu, H. Wu, X. Zhao, L. Yin, T. Jin, X. Liang, J. Tang, Oriented-structured CsCu_2_I_3_ film by close-space sublimation and nanoscale seed screening for high-resolution X-ray imaging. Nano Lett. 21, 1392–1399 (2021). 33480701 10.1021/acs.nanolett.0c04197

[27] R. M. Ribeiro, J. Coutinho, V. J. B. Torres, R. Jones, S. J. Sque, S. Öberg, M. J. Shaw, P. R. Briddon, Ab initio study of CsI and its surface. Phys. Rev. B 74, 035430 (2006).

[28] H. Nishimura, M. Sakata, T. Tsujimoto, M. Nakayama, Origin of the 4.1-eV luminescence in pure CsI scintillator. Phys. Rev. B 51, 2167–2172 (1995). 10.1103/physrevb.51.2167 9978963

[29] A. J. J. Bos, Thermoluminescence as a research tool to investigate luminescence mechanisms. Materials 10, 1357 (2017). 29186873 10.3390/ma10121357 PMC5744292

[30] R. Chen, Glow curves with general order kinetics. J. Electrochem. Soc. 116, 1254 (1969).

[31] Y. Zhou, L. Zhao, Z. Ni, S. Xu, J. Zhao, X. Xiao, J. Huang, Heterojunction structures for reduced noise in large-area and sensitive perovskite x-ray detectors. Sci. Adv. 7, eabg6716 (2021). 34516903 10.1126/sciadv.abg6716 PMC8442865

[32] Y. Gao, Y. Ge, X. Wang, J. Liu, W. Liu, Y. Cao, K. Gu, Z. Guo, Y. Wei, N. Zhou, D. Yu, H. Meng, X. F. Yu, H. Zheng, W. Huang, J. Li, Ultrathin and ultrasensitive direct X-ray detector based on heterojunction phototransistors. Adv. Mater. 33, 2101717 (2021). 10.1002/adma.202101717 34219296

[33] I. Clairand, J. M. Bordy, E. Carinou, J. Daures, J. Debroas, M. Denozire, L. Donadille, M. Ginjaume, C. Itié, C. Koukorava, S. Krim, A. L. Lebacq, P. Martin, L. Struelens, M. Sans-Merce, F. Vanhavere, Use of active personal dosemeters in interventional radiology and cardiology: Tests in laboratory conditions and recommendations–ORAMED project. Radiat. Meas. 46, 1252–1257 (2011).

[34] J. X. Wang, L. Gutiérrez-Arzaluz, X. Wang, T. He, Y. Zhang, M. Eddaoudi, O. M. Bakr, O. F. Mohammed, Heavy-atom engineering of thermally activated delayed fluorophores for high-performance X-ray imaging scintillators. Nat. Photon. 16, 869–875 (2022).

[35] Z. Wang, R. Sun, N. Liu, H. Fan, X. Hu, D. Shen, Y. Zhang, H. Liu, X-Ray imager of 26-μm resolution achieved by perovskite assembly. Nano Res. 15, 2399–2404 (2022).

[36] H. Zhang, Z. Yang, M. Zhou, L. Zhao, T. Jiang, H. Yang, X. Yu, J. Qiu, Y. Yang, X. Xu, Reproducible X-ray imaging with a perovskite nanocrystal scintillator embedded in a transparent amorphous network structure. Adv. Mater. 33, 2102529 (2021). 10.1002/adma.202102529 34418177

[37] X. Ou, X. Qin, B. Huang, J. Zan, Q. Wu, Z. Hong, L. Xie, H. Bian, Z. Yi, X. Chen, Y. Wu, X. Song, J. Li, Q. Chen, H. Yang, X. Liu, High-resolution X-ray luminescence extension imaging. Nature 590, 410–415 (2021). 33597760 10.1038/s41586-021-03251-6

[38] A. Howansky, A. Mishchenko, A. R. Lubinsky, W. Zhao, Comparison of CsI:Tl and Gd_2_O_2_S:Tb indirect flat panel detector X-ray imaging performance in front- and back-irradiation geometries. Med. Phys. 46, 4857–4868 (2019). 31461532 10.1002/mp.13791 PMC6842040

[39] V. Nagarkar, V. Gaysinskiy, Multi-layer radiation detector and related methods. U.S. Patent, US7772558B1 (2010).

[40] A. C. Konstantinidis, M. B. Szafraniec, R. D. Speller, A. Olivo, The Dexela 2923 CMOS X-ray detector: A flat panel detector based on CMOS active pixel sensors for medical imaging applications. Nucl. Inst. Methods Phys. Res. A 689, 12–21 (2012).

[41] N. Haouchine, P. Juvekar, X. Xiong, J. Luo, T. Kapur, R. Du, A. Golby, S. Frisken, Estimation of High Framerate Digital Subtraction Angiography Sequences at Low Radiation Dose, paper presented at the 24th International Conference on Medical Image Computing and Computer Assisted Intervention (Strasbourg, France, 27 September 2021). 10.1007/978-3-030-87231-1_17 PMC8938707 35321151

[42] J. P. Perdew, K. Burke, M. Ernzerhof, Generalized gradient approximation made simple. Phys. Rev. Lett. 77, 3865–3868 (1996). 10062328 10.1103/PhysRevLett.77.3865

[43] P. E. Blöchl, Projector augmented-wave method. Phys. Rev. B 50, 17953–17979 (1994). 10.1103/physrevb.50.17953 9976227

[44] V. Kocevski, Temperature dependence of radiative lifetimes, optical and electronic properties of silicon nanocrystals capped with various organic ligands. J. Chem. Phys. 149, 054301 (2018). 30089370 10.1063/1.5039281

[45] J. Y. Li, C. F. Wang, H. Wu, L. Liu, Q. L. Xu, S. Y. Ye, L. Tong, X. Chen, Q. Gao, Y. L. Hou, F. M. Wang, J. Tang, L. Z. Chen, Y. Zhang, Eco-friendly and highly efficient light-emission ferroelectric scintillators by precise molecular design. Adv. Funct. Mater. 31, 2102848 (2021).

[46] R. Kentsch, M. Morgenroth, M. Scholz, K. Xu, J. Schmedt auf der Günne, T. Lenzer, K. Oum, Direct observation of the exciton self-trapping process in CsCu_2_I_3_ thin films. J. Phys. Chem. Lett. 11, 4286–4291 (2020). 32407630 10.1021/acs.jpclett.0c01130

[47] M. H. Du, Emission trend of multiple self-trapped excitons in luminescent 1D copper halides. ACS Energy Lett. 5, 464–469 (2020).

[48] T. D. Creason, T. M. McWhorter, Z. Bell, M. H. Du, B. Saparov, K_2_CuX_3_(X = Cl, Br): All-inorganic lead-free blue emitters with near-unity photoluminescence quantum yield. Chem. Mater. 32, 6197–6205 (2020).

[49] L. Stand, D. Rutstrom, M. Koschan, M. H. Du, C. Melcher, U. Shirwadkar, J. Glodo, E. Van Loef, K. Shah, M. Zhuravleva, Crystal growth and scintillation properties of pure and Tl-doped Cs_3_Cu_2_I_5_. Nucl. Inst. Methods Phys. Res. A 991, 164963 (2021).

[50] J. Tauc, A. Menth, States in the gap. J. Non Cryst. Solids 8, 569–585 (1972).

[51] S. Tavernier, A. Gektin, B. Grinyov, W. W. Moses, *Radiation Detectors for Medical Applications* (Springer, 2013).

[52] J. A. Shepherd, Study of afterglow in X-ray phosphors for use on fast-framing charge-coupled device detectors. Opt. Eng. 36, 3212 (1997).

[53] W. J. C. Koppert, M. M. A. Dietze, S. van Der Velden, J. H. L. Steenbergen, H. W. A. M. de Jong, A comparative study of NaI(Tl), CeBr_3_, and CZT for use in a real-time simultaneous nuclear and fluoroscopic dual-layer detector. Phys. Med. Biol. 64, 135012 (2019). 31158823 10.1088/1361-6560/ab267c

[54] V. V. Nagarkar, S. Miller, B. Singh, S. Thacker, V. Gaysinskiy, B. W. Meller, H. B. Barber, D. Wilson, Development of microcolumnar LaBr_3_:Ce scintillator. Penetrating Radiat. Syst. Appl. X 7450, 745006 (2009).

[55] S. Cheng, M. Nikl, A. Beitlerova, R. Kucerkova, X. Du, G. Niu, Y. Jia, J. Tang, G. Ren, Y. Wu, Ultrabright and highly efficient all-inorganic zero-dimensional perovskite scintillators. Adv. Opt. Mater. 9, 2100460 (2021).

[56] S. Cheng, A. Beitlerova, R. Kucerkova, E. Mihokova, M. Nikl, Z. Zhou, G. Ren, Y. Wu, Non-hygroscopic, self-absorption free, and efficient 1D CsCu_2_I_3_ perovskite single crystal for radiation detection. ACS Appl. Mater. Interfaces 13, 12198–12202 (2021). 33656315 10.1021/acsami.0c22505

[57] Y. Wei, Z. Cheng, J. Lin, An overview on enhancing the stability of lead halide perovskite quantum dots and their applications in phosphor-converted LEDs. Chem. Soc. Rev. 48, 310–350 (2019). 30465675 10.1039/c8cs00740c

