# Supplementary material for: One-dimensional scintillator film with benign grain boundaries for high-resolution and fast x-ray imaging
Source: Sci Adv. 2023 Jul 28;9(30):eadh1789. doi: 10.1126/sciadv.adh1789 (PMC10381942; doi:10.1126/sciadv.adh1789)
Supplement: Supplementary file 1 — Supplementary Text Figs. S1 and S27 Tables S1 to S4 Legends for movies S1 and S2 References [file sciadv.adh1789_sm.pdf]

Science Advances
AAAS

Supplementary Materials for

One-dimensional scintillator film with benign grain boundaries for high-resolution and fast x-ray imaging

Haodi Wu *et al.*

Corresponding author: Guangda Niu, guangda_niu@hust.edu.cn; Yuntao Wu, ytwu@mail.sic.ac.cn; Jiang Tang, jtang@mail.hust.edu.cn

Sci. Adv. **9**, eadh1789 (2023)
DOI: 10.1126/sciadv.adh1789

The PDF file includes:

Supplementary Text
Figs. S1 and S27
Tables S1 to S4
Legends for movies S1 and S2
References

Other Supplementary Material for this manuscript includes the following:

Movies S1 and S2

Supplementary Text

The origin of STE1 and STE2 in $Cs_5Cu_3Cl_6I_2$

Under UV light excitation, in $Cs_5Cu_3Cl_6I_2$, electrons in the valence band (Cu 3d) are excited to the conduction band (Cu 4s), and leave holes in the valence band. The e-h pairs attract each other due to the Coulomb force, and form free excitons over a short time range (~ps) (*46*). Due to the distortions of tetrahedral units and 1D zigzag chains, subsequently, the free excitons are further trapped near the Cu ions and form STEs. Because the valence and conduction bands mainly derived from Cu ions, the exciton energy can be lowered when two or more adjacent Cu ions move closer to enhance the hybridization between the Cu-4s orbitals (*47*). This process usually involves with the breaking of Cu-X bonds and the formation of Cu-Cu bonds (*47–49*). In $CsCu_2X_3$, there are three different types of Cu-Cu bonds based on the theoretical calculations, but only one STE emission was experimentally observed due to the halogen-dependent relative stabilities of three type of STEs *(47)*. As observed in fig. S15F, in $Cs_5Cu_3Cl_6I_2$, there are two different types of Cu-Cu bond, namely Cu1-Cu2 (from the edge-shared two tetrahedral units) and Cu1(or Cu2)-Cu3 (from one of the edge-shared two tetrahedral units and the third vertex-shared single tetrahedral units). The lattice deformation energy (E_d) can be calculated according to the general description of STE emission (*47*):

$$E_{pl} = E_g - E_a - E_d$$

where E_{pl} is the emission energy of STE1 and STE2, E_g is the band-gap, and E_a is the STE binding energy (1087 meV for STE1 and 395 meV for STE2). Using the available values of E_{pl} and E_g, the E_d of STE1 was lower than that of STE2 (E_{d2}-E_{d1}=1.17 eV). It suggests that STE1 has a weaker distortion than STE2. The vertex-shared units are generally easier to tilt, twist and distortion than the edge-shared units, in particular for the zigzag chains. Thus, the formation of STE1 can be correlated to the deformation of Cu1-Cu2 bonds, and STE2 to Cu1(or Cu2)-Cu3 bonds.

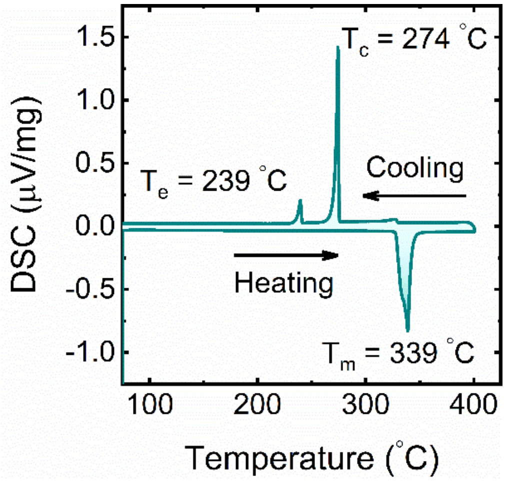

Fig. S1. DSC curve of a $Cs_5Cu_3Cl_6I_2$ single crystal.

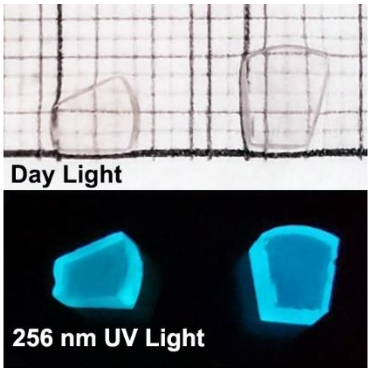

Fig. S2. As-polished $Cs_5Cu_3Cl_6I_2$ crystals. The crystals are transparent, colorless, and inclusion-free. Under 256 nm UV light excitation, the crystals emit bright cyan light.

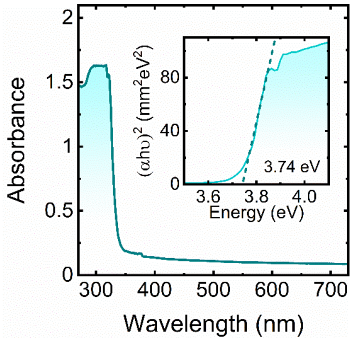

Fig. S3. Optical absorption spectrum of the $Cs_5Cu_3Cl_6I_2$. The optical band gap is estimated by using the Tauc's model (*50*): $(\alpha h\nu)^{1/n}=A(h\nu-E_g)$, where α is the absorption coefficient, $h\nu$ is the photon energy, E_g is the optical energy gap, and the constant A is the band tailing parameter. The index n is related to the type of band gap of the material, which is 1/2, 2, 3/2 and 3 for the direct allowed, indirect allowed, direct forbidden and indirect forbidden transition, respectively. Since the $Cs_5Cu_3Cl_6I_2$ is allowed direct transition *(24)*, the value n is 1/2. Thus, the optical band gap of $Cs_5Cu_3Cl_6I_2$ is estimated as 3.74 eV.

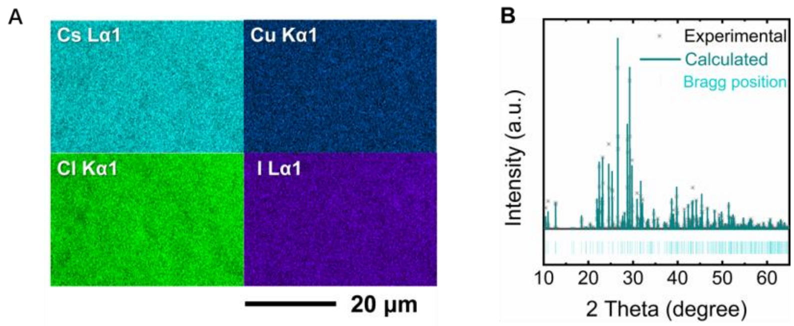

Fig. S4. EDS elemental mapping and XRD of the as grew $Cs_5Cu_3Cl_6I_2$. (**A**) The EDS elemental mapping of the as grew $Cs_5Cu_3Cl_6I_2$, which displays homogeneous distributions of the cesium (Cs), copper (Cu), chloride (Cl), and iodine (I) elements in the $Cs_5Cu_3Cl_6I_2$ crystal. (**B**) Rietveld refinement of the XRD data for $Cs_5Cu_3Cl_6I_2$. The black symbols represent the experimental data, and the green line is the simulated diffractogram. The cyan short lines are associated to the Bragg reflection positions.

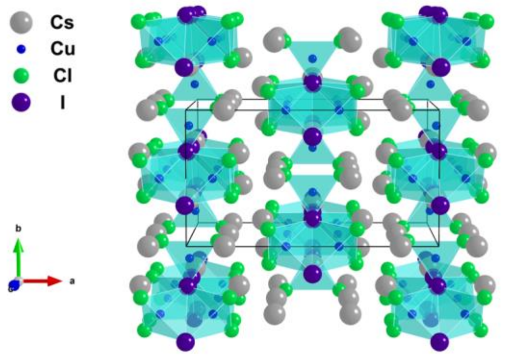

Fig. S5. Side view of crystal structure of $Cs_5Cu_3Cl_6I_2$ along the c-axis.

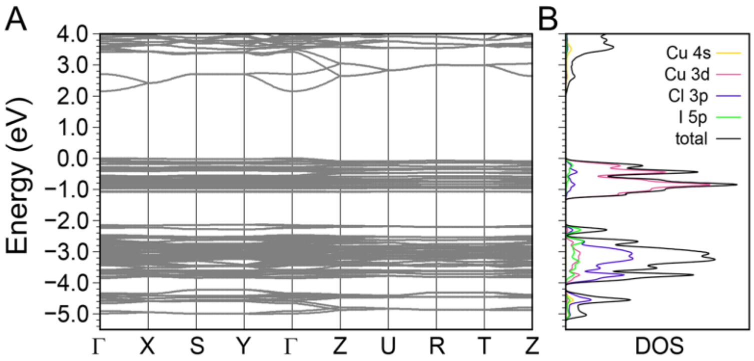

Fig. S6. Calculated electronic band structures and density of states (DOS). (**A**) Band structure of $Cs_5Cu_3Cl_6I_2$. (**B**) Total density of states (DOS) and partial DOS of Cu 4s, Cu 3d, Cl 3p and I 5p orbitals.

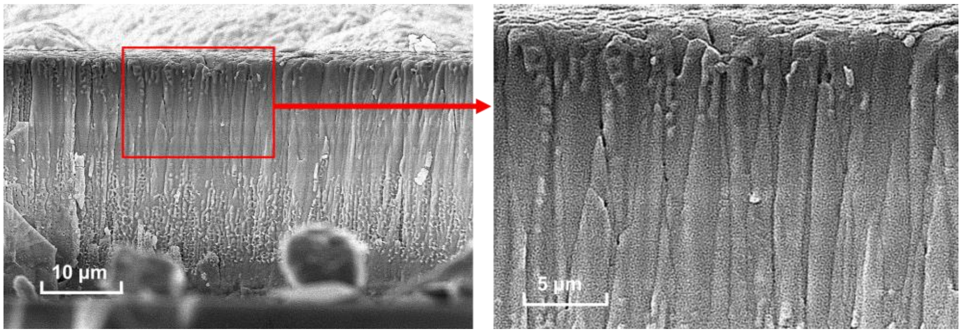

Fig. S7. Columnar crystal structure of the as fabricated $Cs_5Cu_3Cl_6I_2$ film.

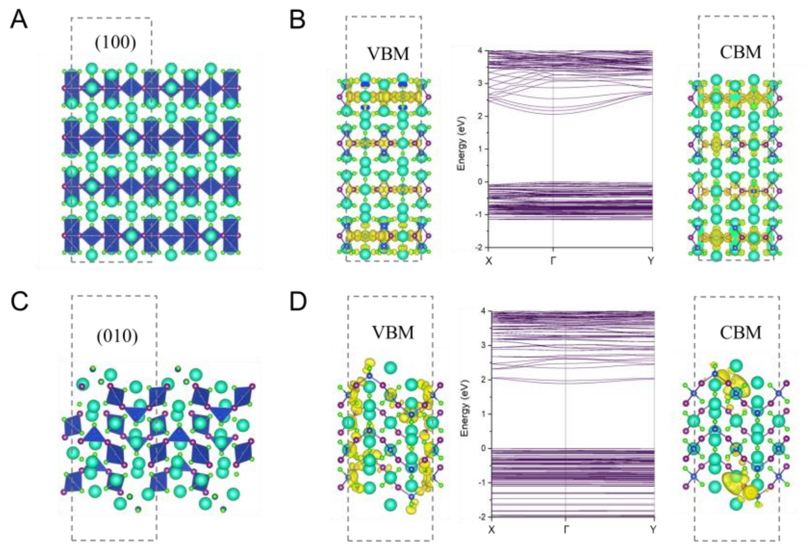

Fig. S8. Calculated atomic and electronic structures of different surfaces. (**A**) Atomic structure of (100) surface and (**B**) the corresponding band structure, electronic charge density at valence band minimum (VBM) and conduction band minimum (CBM). (**C**) and (d) show the cases corresponding to (010) surface.

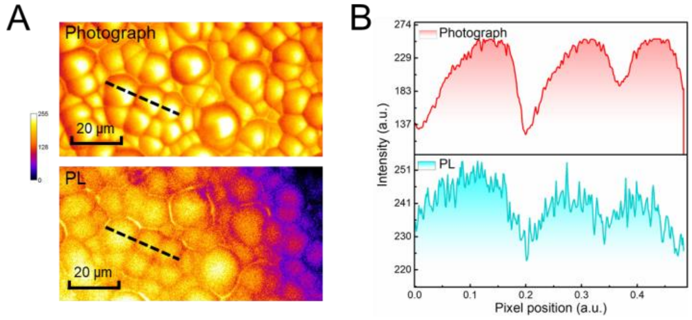

Fig. S9. PL mapping of CsI: Tl film. (A) Two-dimensional topography optical microscope image and PL map of CsI: Tl film. (B) Intensity of the illustrative line in (A). The grey value of PL map shows that the luminous intensity is reduced obviously at GBs.

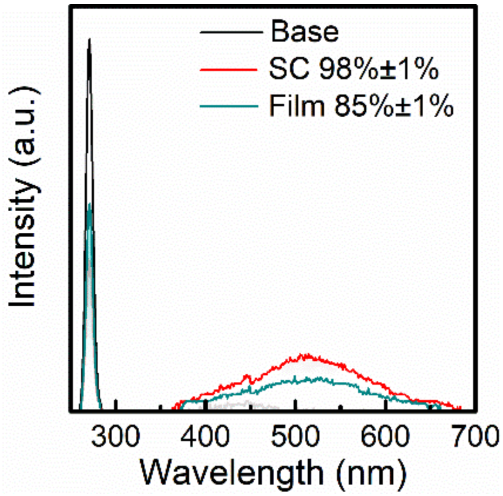

Fig. S10. PLQY of CsI: Tl single crystal and film.

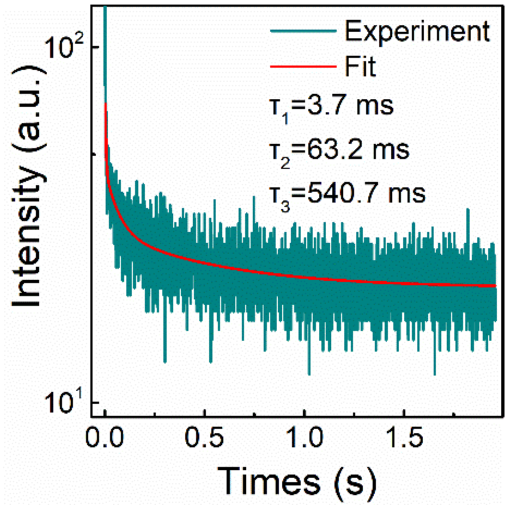

Fig. S11. PL decay of CsI:Tl film and fit by sum of three-exponentials. The excitation wavelength is 292 nm and the recorded wavelength is 550 nm.

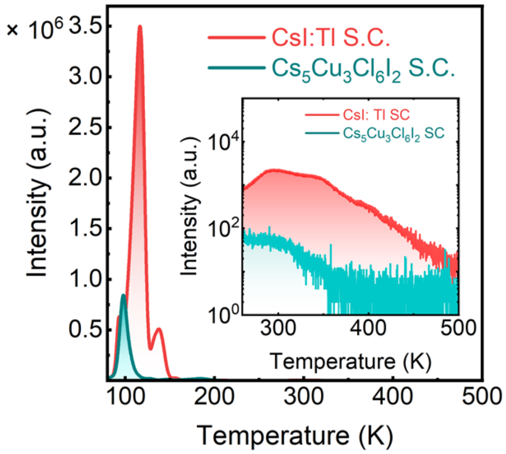

Fig. S12. TSL measurement. TSL measurement of $Cs_5Cu_3Cl_6I_2$ single crystal (cyan color) and CsI:Tl single crystal (red color) with the same size. Inset is the comparison of 260-500 K in logarithmic coordinates.

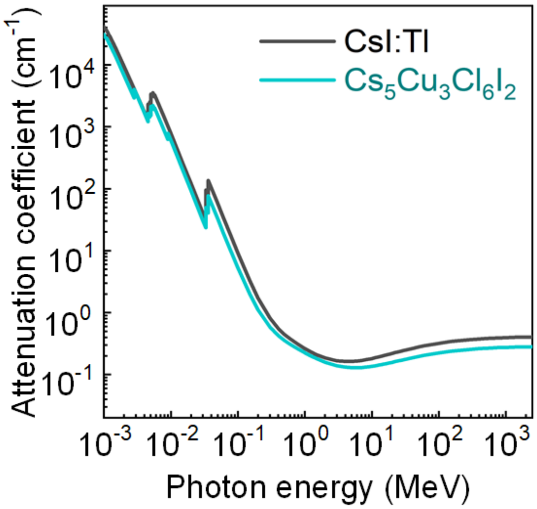

Fig. S13. Attenuation coefficients calculated in a broad energy range of X(γ)-ray photons for $Cs_5Cu_3Cl_6I_2$ and CsI: Tl.

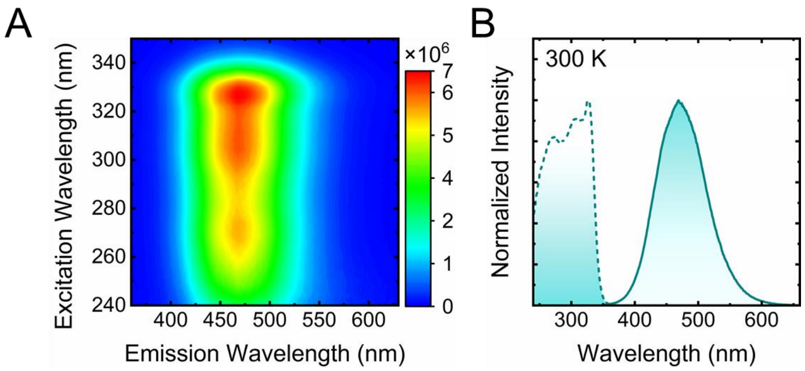

Fig. S14. PL and PLE of the $Cs_5Cu_3Cl_6I_2$. (**A**) PL excitation and emission contour mapping of $Cs_5Cu_3Cl_6I_2$ at RT. (**B**) PL and PLE spectra at RT. The emission peaks at 470 nm with a FWHM of 90 nm. The Stokes shift is 1.18 eV, without any self-absorption.

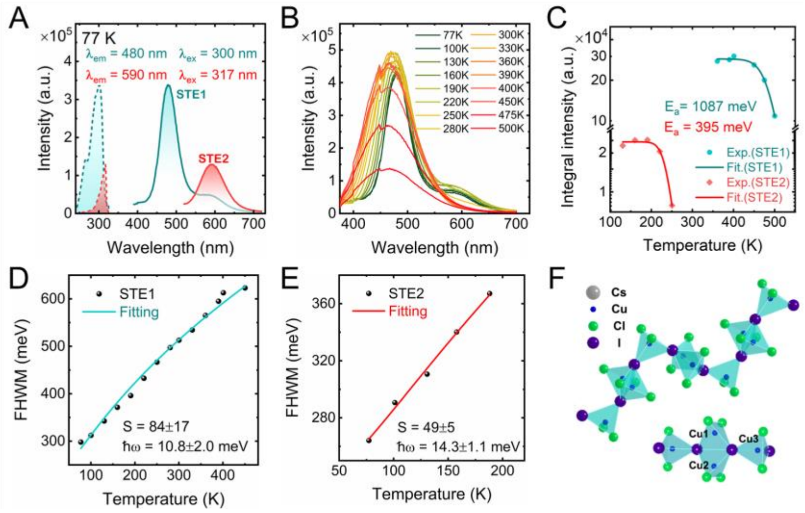

Fig. S15. Physical mechanism of STE emission. (**A**) PL and PLE spectra of two STE emissions at 77 K. Note that there are two emission centers (480 nm, STE1 and 590 nm, STE2) under 77 K, which can be correlated with the deformation of different Cu-Cu bonds in the zigzag chains. (**B**) Temperature dependent PL spectra. The onset of thermal quenching occurs around 400 K for the STE1 emission, and around 200 K for the STE2 emission. (**C**) Integrated PL intensity of STE1 and STE2 as a function of temperature. The activation energy of emission quenching is estimated as 1087 meV for STE1 and 395 meV for STE2. (**D**), (**E**) The temperature dependent FWHM as a function of temperature for (D) STE1 and (E) STE2. (**F**) The schematic view of $[Cu_3Cl_6I_2]_n^{5n-}$ basic units.

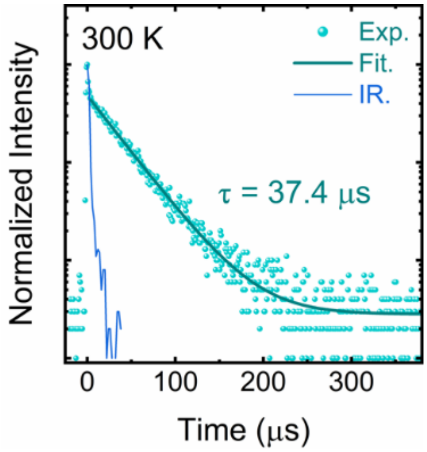

Fig. S16. PL decay profile of $Cs_5Cu_3Cl_6I_2$ at RT. The excitation wavelength is 290 nm and the recorded wavelength is 479 nm.

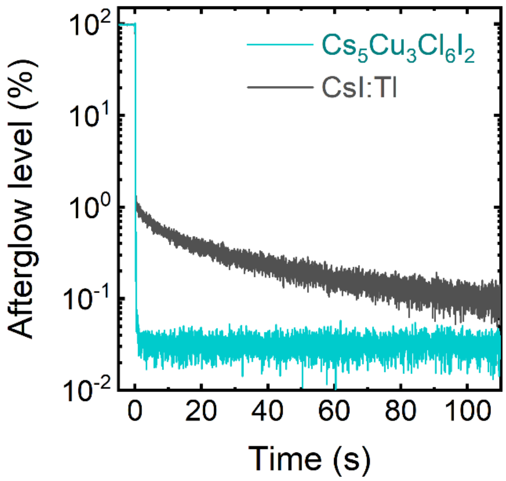

Fig. S17. X-ray induced afterglow profiles of $Cs_5Cu_3Cl_6I_2$ and CsI:Tl films in an extended time scale.

A

B

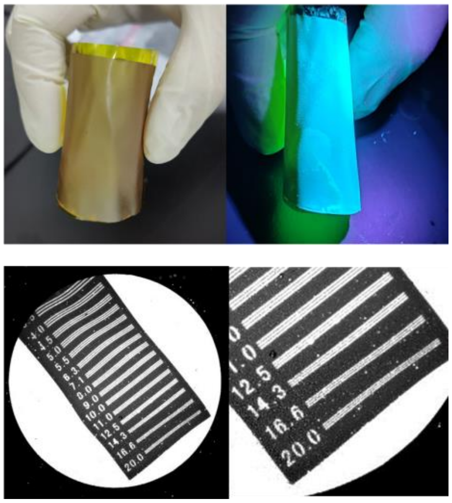

Fig. S18. Flexible $Cs_5Cu_3Cl_6I_2$ film and its X-ray imaging. (**A**) The photograph of the fabricated $Cs_5Cu_3Cl_6I_2$ film on a polyimide substrate under day light and under UV light excitation. (**B**) X-ray imaging of a standard X-ray resolution pattern plate by using flexible $Cs_5Cu_3Cl_6I_2$ film.

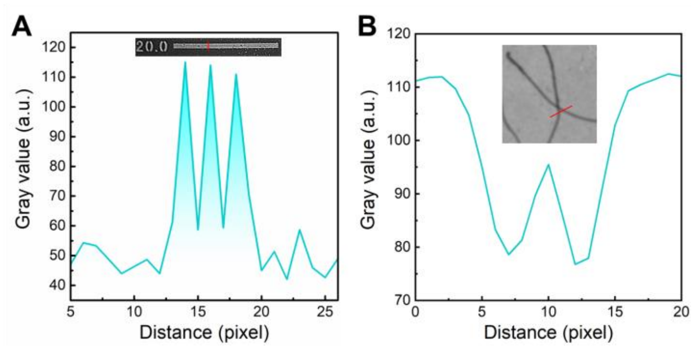

Fig. S19. The change of gray value for line pair card at 20 lp mm^{-1} and gold wires with 15 μm diameter. The MTF is calculated as 0.317 and 0.103, which is consistent with the MTF curve.

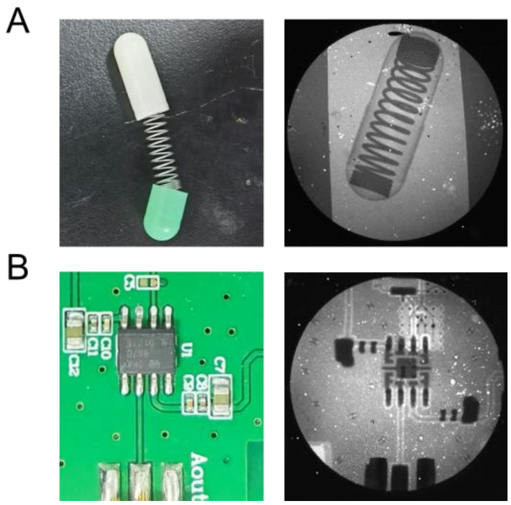

Fig. S20. X-ray imaging. (A and **B)** Photograph and X-ray image of (A) a spring and (B) PCB.

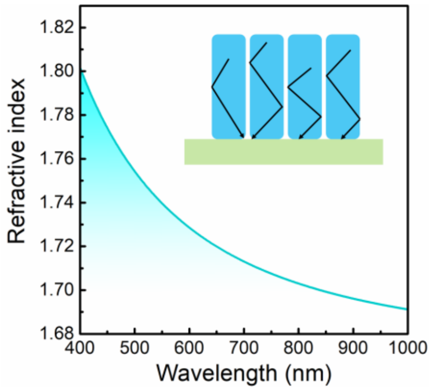

Fig. S21. Refractive index versus wavelength of the $Cs_5Cu_3Cl_6I_2$.

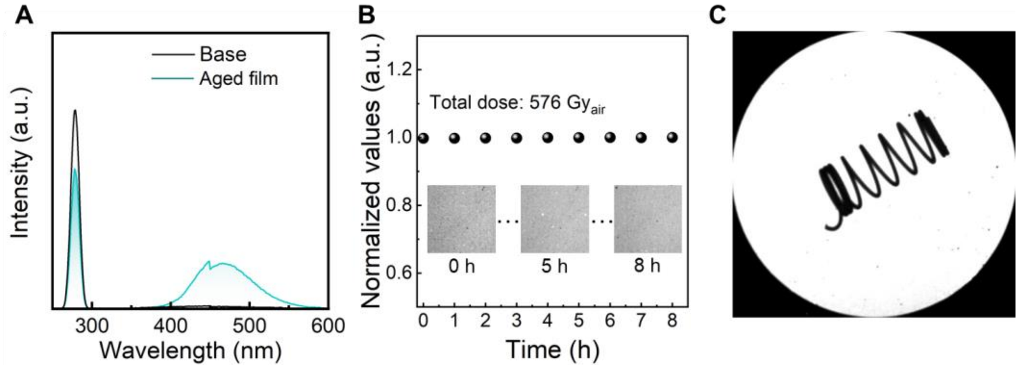

Fig. S22. Stability of the $Cs_5Cu_3Cl_6I_2$ film. (A) The PLQY of Cs5Cu3Cl6I2 film remained at 92% after placed in the air for 3 months. (B) The output pixel values of $Cs_5Cu_3Cl_6I_2$ film under continuous X-ray irradiation. The inset shows the X-ray bright field imaging of the $Cs_5Cu_3Cl_6I_2$ film after different times of irradiation. (C) X-ray imaging of a spring using aged $Cs_5Cu_3Cl_6I_2$ film.

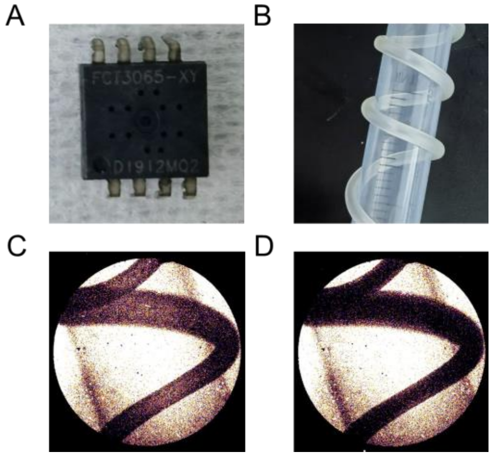

Fig. S23. Demonstration of chip inspection and angiography. (**A**) Photograph of the chip for the demonstration of pipelining inspection. (**B**) Photograph of the hose, which was used to simulate a blood vessel. (**C** and **D**) The X-ray image of (B) without (C) and with (D) iodinated contrast media injection.

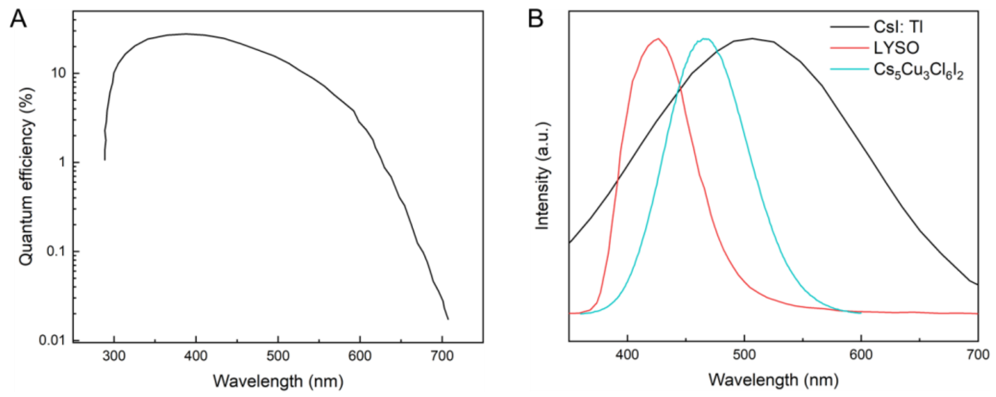

Fig. S24. Details of scintillation yield calculation. (**A**) The PMT spectral quantum efficiency. (**B**) The RL spectrum of CsI: Tl, LYSO and $Cs_5Cu_3Cl_6I_2$ under X-ray excitation.

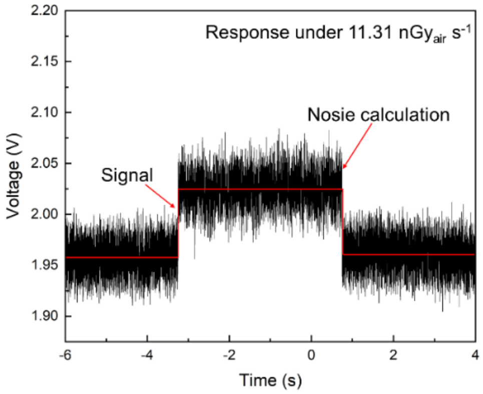

Fig. S25. Scintillator response under 11.31 nGy_{air} s^{-1} by tuning the X-ray tube on and off. The red line is the average value of dark voltage and photovoltage.

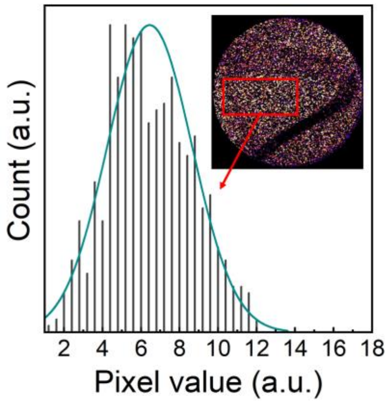

Fig. S26. The scintillation photon noise and the camera's electronic noise of the angiography image. The noise follows a Gaussian distribution, with an average pixel value of 6.4.

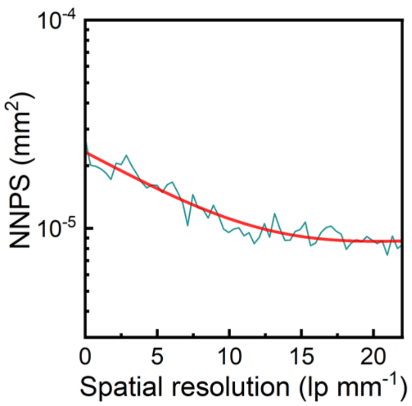

Fig. S27. The normalize noise power spectrum (NPS) of the $Cs_5Cu_3Cl_6I_2$ film X-ray imager.

Table S1. Performance comparison of scintillators.

Scintillator	Light yield (p/MeV)	Afterglow	Columnar structure	Stability	Non-toxicity	Non-radioactive	Ref
**CsI: Tl SC**	54000	Severe (>2%@3 ms)	NA	Hygroscopic	× (Tl)	√	(*12*, *51*)
**CsI: Tl film**	48000	Severe (4%@3 ms)	√	Hygroscopic	× (Tl)	√	(*8*, *17*)
**Gd_2O_2S: Tb**	60000	Negligible (0.6%@3 ms)	×	Good	√	√	(*12*, *52*)
**$CdWO_4$**	6200-28000	Negligible (<0.1%@3 ms)	×	Good	× (Cd)	√	(*12*)
**NaI: Tl**	38000	Severe (14.1%@3 ms)	×	Strongly hygroscopic	× (Tl)	√	(*51*, *53*)
**$LaBr_3$: Ce**	61000	Negligible (0.3%@180 ns)	×	Very strongly hygroscopic	√	× (La)	(*12*, *54*)
**BGO**	9000	Negligible (0.01%@20 ms)	×	Good	√	√	(*12*)
**$Cs_3Cu_2I_5$**	32000	Negligible (0.03%@10 ms)	×	Good	√	√	(*55*)
**$CsCu_2I_3$**	21580	Negligible (0.008%@10 ms)	√	Good	√	√	(*26*, *56*)
**Rb_2CuBr_3**	91056	NA	×	Good	√	× (Rb)	(*21*)
**$CsPbBr_3$ QDs**	35676	NA	×	Hygroscopic/ Thermal degradation	× (Pb)	√	(*10*, *57*)
**$NaLuF_4$: Tb @ $NaYF_4$ NPs**	NA	Severe (>60%@ 1200 s)	×	Good	√	× (Lu)	(*37*)
**$Cs_5Cu_3Cl_6I_2$**	**65000**	**Negligible (0.1%@10 ms)**	√	**Good**	√	√	**This work**

Table S2. X-ray diffraction data and collection parameters of $Cs_5Cu_3Cl_6I_2$.

Chemical formula	$Cs_5Cu_3Cl_6I_2$
Formula weight	1321.67
Temperature	293(2) K
Radiation, wavelength	Mo Kα, 0.71073 Å
Crystal system	Orthorhombic
Space group	Cmcm
Z	4
Unit cell parameters	a=16.9110 Å, α=90°<br>b=9.1470 Å, β=90°<br>c=14.0570 Å, γ=90°
Volume	2174.6 $Å^3$
Density (calculated)	4.037 g/cm^3
Absorption coefficient	14.709 mm^{-1}
F(000)	2280
θ range for data collection	2.409° to 26.367°
Reflections collected	49659
Independent reflections	1084
Data/restraints/parameters	1197/0/47
Goodness-of-fit on F^2	1.083
Final R indexes [I > 2σ (I)]	R_{obs}=0.0179,<br>wR_{obs}=0.0381
Final R indexes [all data]	R_{all}=0.0210, wR_{all}=0.0392

Table S3. Performance comparison of scintillators.

	Peak (K)	Trap depth (eV)	Frequency factor (s^{-1})	De-trapping time
$Cs_5Cu_3Cl_6I_2$ SC	106	0.28	10^{12}	59.0 ns
	118	0.31	10^{12}	188 ns
	125	0.33	10^{12}	408 ns
	134	0.36	10^{12}	1.03 μs
	147	0.39	10^{12}	4.16 μs
	166	0.45	10^{12}	32.3 μs
	209	0.57	10^{12}	3.10 ms
$Cs_5Cu_3Cl_6I_2$ Film	106	0.28	10^{12}	50.6 ns
	114	0.30	10^{12}	118 ns
	120	0.32	10^{12}	238 ns
	128	0. 34	10^{12}	535 ns
	137	0.37	10^{12}	1.46 μs
	177	0.47	10^{12}	99.2 μs
	219	0.60	10^{12}	5.26 ms

Table S4. Physical properties of $Cs_5Cu_3Cl_6I_2$ and CsI: Tl.

Composition	$Cs_5Cu_3Cl_6I_2$	CsI:Tl
Density (g/cm^3)	3.8	4.5
Z_{eff}	49	54
Melting point (°C)	339	620
Hygroscopic	No	Slight
Emission wavelength (nm)	470	550

Movie S1 demonstration of chip inspection.

Movie S2 demonstration of angiography.

REFERENCES AND NOTES

1. P. Russo, Ed., in *Handbook of X-ray Imaging: Physics and Technology* (CRC Press, 2017), p. 1419.

2. K. Chrzanowski, Review of night vision metrology. *Opto-Electronics Rev.* **23**, 149–164 (2015).

3. R. Autrata, P. Schauer, J. Kuapil, J. Kuapil, A single crystal of YAG-new fast scintillator in SEM. *J. Phys. E* **11**, 707–708 (1978).

4. E. Auffray, F. Cavallari, M. Lebeau, P. Lecoq, M. Schneegans, P. Sempere-Roldan, Crystal conditioning for high-energy physics detectors. *Nucl. Inst. Methods Phys. Res. A* **486**, 22–34 (2002).

5. C. Dujardin, E. Auffray, E. Bourret-Courchesne, P. Dorenbos, P. Lecoq, M. Nikl, A. N. Vasil'Ev, A. Yoshikawa, R. Y. Zhu, Needs, trends, and advances in inorganic scintillators. *IEEE Trans. Nucl. Sci.* **65**, 1977–1997 (2018).

6. T. Martin, A. Koch, Recent developments in X-ray imaging with micrometer spatial resolution. *J. Synchrotron Radiat.* **13**, 180–194 (2006).

7. V. V. Nagarkar, T. K. Gupta, S. R. Miller, Y. Klugerman, M. R. Squillante, G. Entine, Structured CsI(Tl) scintillators for X-ray imaging applications. *IEEE Trans. Nucl. Sci.* **45**, 492–496 (1998).

8. V. V. Nagarkar, S. C. Thacker, V. Gaysinskiy, L. E. Ovechkina, S. R. Miller, S. Cool, C. Brecher, Suppression of afterglow in microcolumnar CsI:Tl by codoping with Sm: Recent advances. *IEEE Trans. Nucl. Sci.* **56**, 565–569 (2009).

9. Y. Wu, G. Ren, F. Meng, X. Chen, D. Ding, H. Li, S. Pan, Effects of Bi^{3+} codoping on the optical and scintillation properties of CsI:Tl single crystals. *Phys. Status Solidi* **211**, 2586–2591 (2014).

10. Q. Chen, J. Wu, X. Ou, B. Huang, J. Almutlaq, A. A. Zhumekenov, X. Guan, S. Han, L. Liang, Z. Yi, J. Li, X. Xie, Y. Wang, Y. Li, D. Fan, D. B. L. Teh, A. H. All, O. F. Mohammed, O. M. Bakr, T. Wu, M. Bettinelli, H. Yang, W. Huang, X. Liu, All-inorganic perovskite nanocrystal scintillators. *Nature* **561**, 88–93 (2018).

11. C. Greskovich, S. Duclos, Ceramic scintillators. *Annu. Rev. Mater. Sci.* **27**, 69–88 (1997).

12. C. W. E. van Eijk, Inorganic scintillators in medical imaging detectors. *Nucl. Inst. Methods Phys. Res. A* **509**, 17–25 (2003).

13. G. Blasse, B. C. Grabmaier, *Luminescent Materials* (Springer, 1994).

14. D. Chiriu, N. Faedda, A. G. Lehmann, P. C. Ricci, A. Anedda, S. Desgreniers, E. Fortin, Structural characterization of $Lu_{1.8}Y_{0.2}SiO_5$ crystals. *Phys. Rev. B - Condens. Matter Mater. Phys.* **76**, 054112 (2007).

15. L. Labr, K. Kramer, M. Schulze, Three bromides of lanthanum: $LaBr_2$, La_2Br_5, and $LaBr_3$. *Z. Anorg. Allg. Chem.* **575**, 61–70 (1989).

16. Y. Zorenko, T. Voznyak, R. Turchak, A. Fedorov, K. Wiesniewski, M. Grinberg, Luminescent and scintillation properties of CsI:Tl films grown by the liquid phase epitaxy method. *Phys. Status Solidi Appl. Mater. Sci.* **207**, 2344–2350 (2010).

17. A. Fedorov, A. Lebedinsky, O. Zelenskaya, Scintillation efficiency, structure and spatial resolution of CsI(Tl) layers. *Nucl. Instr. Meth. Phys. Res. A* **564**, 328–331 (2006).

18. M. Chhowalla, D. Jena, H. Zhang, Two-dimensional semiconductors for transistors. *Nat. Rev. Mater.* **1**, 16052 (2016).

19. R. Lin, Q. Guo, Q. Zhu, Y. Zhu, W. Zheng, F. Huang, All-inorganic $CsCu_2I_3$ single crystal with high-PLQY (≈15.7%) intrinsic white-light emission via strongly localized 1D excitonic recombination. *Adv. Mater.* **31**, 1905079 (2019).

20. M. Zhang, X. Wang, B. Yang, J. Zhu, G. Niu, H. Wu, L. Yin, X. Du, M. Niu, Y. Ge, Q. Xie, Y. Yan, J. Tang, Metal halide scintillators with fast and self-absorption-free defect-bound excitonic radioluminescence for dynamic X-ray imaging. **31**, 2007921 (2021).

21. B. Yang, L. Yin, G. Niu, J. Yuan, K. Xue, Z. Tan, X. Miao, M. Niu, X. Du, H. Song, E. Lifshitz, J. Tang, Lead-free halide Rb_2CuBr_3 as sensitive X-ray scintillator. *Adv. Mater.* **31**, e1904711 (2019).

22. M. Baskaran, *Handbook of Environmental Isotope Geochemistry* (Springer, 2012).

23. S. O. Ferreira, *Advanced Topics on Crystal Growth* (InTech, 2013).

24. J. Li, T. Inoshita, T. Ying, A. Ooishi, J. Kim, H. Hosono, A highly efficient and stable blue-emitting $Cs_5 Cu_3 Cl_6 I_2$ with a 1D chain structure. *Adv. Mater.* **32**, 2002945 (2020).

25. X. Niu, J. Xiao, B. Lou, Z. Yan, Q. Zhou, T. Lin, C. Ma, X. Han, Highly efficient blue emissive copper halide $Cs_5Cu_3Cl_6I_2$ scintillators for X-ray detection and imaging. *Ceram. Int.* **48**, 30788–30796 (2022).

26. M. Zhang, J. Zhu, B. Yang, G. Niu, H. Wu, X. Zhao, L. Yin, T. Jin, X. Liang, J. Tang, Oriented-structured $CsCu_2I_3$ film by close-space sublimation and nanoscale seed screening for high-resolution X-ray imaging. *Nano Lett.* **21**, 1392–1399 (2021).

27. R. M. Ribeiro, J. Coutinho, V. J. B. Torres, R. Jones, S. J. Sque, S. Öberg, M. J. Shaw, P. R. Briddon, Ab initio study of CsI and its surface. *Phys. Rev. B* **74**, 035430 (2006).

28. H. Nishimura, M. Sakata, T. Tsujimoto, M. Nakayama, Origin of the 4.1-eV luminescence in pure CsI scintillator. *Phys. Rev. B* **51**, 2167 (1995), 2172.

29. A. J. J. Bos, Thermoluminescence as a research tool to investigate luminescence mechanisms. *Materials* **10**, 1357 (2017).

30. R. Chen, Glow curves with general order kinetics. *J. Electrochem. Soc.* **116**, 1254 (1969).

31. Y. Zhou, L. Zhao, Z. Ni, S. Xu, J. Zhao, X. Xiao, J. Huang, Heterojunction structures for reduced noise in large-area and sensitive perovskite x-ray detectors. *Sci. Adv.* **7**, eabg6716 (2021).

32. Y. Gao, Y. Ge, X. Wang, J. Liu, W. Liu, Y. Cao, K. Gu, Z. Guo, Y. Wei, N. Zhou, D. Yu, H. Meng, X. F. Yu, H. Zheng, W. Huang, J. Li, Ultrathin and ultrasensitive direct X-ray detector based on heterojunction phototransistors. *Adv. Mater.* **33**, 2101717 (2021).

33. I. Clairand, J. M. Bordy, E. Carinou, J. Daures, J. Debroas, M. Denozire, L. Donadille, M. Ginjaume, C. Itié, C. Koukorava, S. Krim, A. L. Lebacq, P. Martin, L. Struelens, M. Sans-Merce, F. Vanhavere, Use of active personal dosemeters in interventional radiology and cardiology: Tests in laboratory conditions and recommendations–ORAMED project. *Radiat. Meas.* **46**, 1252–1257 (2011).

34. J. X. Wang, L. Gutiérrez-Arzaluz, X. Wang, T. He, Y. Zhang, M. Eddaoudi, O. M. Bakr, O. F. Mohammed, Heavy-atom engineering of thermally activated delayed fluorophores for high-performance X-ray imaging scintillators. *Nat. Photon.* **16**, 869–875 (2022).

35. Z. Wang, R. Sun, N. Liu, H. Fan, X. Hu, D. Shen, Y. Zhang, H. Liu, X-Ray imager of 26-μm resolution achieved by perovskite assembly. *Nano Res.* **15**, 2399–2404 (2022).

36. H. Zhang, Z. Yang, M. Zhou, L. Zhao, T. Jiang, H. Yang, X. Yu, J. Qiu, Y. Yang, X. Xu, Reproducible X-ray imaging with a perovskite nanocrystal scintillator embedded in a transparent amorphous network structure. *Adv. Mater.* **33**, 2102529 (2021).

37. X. Ou, X. Qin, B. Huang, J. Zan, Q. Wu, Z. Hong, L. Xie, H. Bian, Z. Yi, X. Chen, Y. Wu, X. Song, J. Li, Q. Chen, H. Yang, X. Liu, High-resolution X-ray luminescence extension imaging. *Nature* **590**, 410–415 (2021).

38. A. Howansky, A. Mishchenko, A. R. Lubinsky, W. Zhao, Comparison of CsI:Tl and Gd_2O_2S:Tb indirect flat panel detector X-ray imaging performance in front- and back-irradiation geometries. *Med. Phys.* **46**, 4857–4868 (2019).

39. V. Nagarkar, V. Gaysinskiy, Multi-layer radiation detector and related methods. U.S. Patent, US7772558B1 (2010).

40. A. C. Konstantinidis, M. B. Szafraniec, R. D. Speller, A. Olivo, The Dexela 2923 CMOS X-ray detector: A flat panel detector based on CMOS active pixel sensors for medical imaging applications. *Nucl. Inst. Methods Phys. Res. A* **689**, 12–21 (2012).

41. N. Haouchine, P. Juvekar, X. Xiong, J. Luo, T. Kapur, R. Du, A. Golby, S. Frisken, Estimation of High Framerate Digital Subtraction Angiography Sequences at Low Radiation Dose, paper presented at the 24th International Conference on Medical Image Computing and Computer Assisted Intervention (Strasbourg, France, 27 September 2021).

42. J. P. Perdew, K. Burke, M. Ernzerhof, Generalized gradient approximation made simple. *Phys. Rev. Lett.* **77**, 3865–3868 (1996).

43. P. E. Blöchl, Projector augmented-wave method. *Phys. Rev. B* **50**, 17953–17979 (1994).

44. V. Kocevski, Temperature dependence of radiative lifetimes, optical and electronic properties of silicon nanocrystals capped with various organic ligands. *J. Chem. Phys.* **149**, 054301 (2018).

45. J. Y. Li, C. F. Wang, H. Wu, L. Liu, Q. L. Xu, S. Y. Ye, L. Tong, X. Chen, Q. Gao, Y. L. Hou, F. M. Wang, J. Tang, L. Z. Chen, Y. Zhang, Eco-friendly and highly efficient light-emission ferroelectric scintillators by precise molecular design. *Adv. Funct. Mater.* **31**, 2102848 (2021).

46. R. Kentsch, M. Morgenroth, M. Scholz, K. Xu, J. Schmedt auf der Günne, T. Lenzer, K. Oum, Direct observation of the exciton self-trapping process in $CsCu_2I_3$ thin films. *J. Phys. Chem. Lett.* **11**, 4286–4291 (2020).

47. M. H. Du, Emission trend of multiple self-trapped excitons in luminescent 1D copper halides. *ACS Energy Lett.* **5**, 464–469 (2020).

48. T. D. Creason, T. M. McWhorter, Z. Bell, M. H. Du, B. Saparov, K_2CuX_3(X = Cl, Br): All-inorganic lead-free blue emitters with near-unity photoluminescence quantum yield. *Chem. Mater.* **32**, 6197–6205 (2020).

49. L. Stand, D. Rutstrom, M. Koschan, M. H. Du, C. Melcher, U. Shirwadkar, J. Glodo, E. Van Loef, K. Shah, M. Zhuravleva, Crystal growth and scintillation properties of pure and Tl-doped $Cs_3Cu_2I_5$. *Nucl. Inst. Methods Phys. Res. A* **991**, 164963 (2021).

50. J. Tauc, A. Menth, States in the gap. *J. Non Cryst. Solids* **8**, 569–585 (1972).

51. S. Tavernier, A. Gektin, B. Grinyov, W. W. Moses, *Radiation Detectors for Medical Applications* (Springer, 2013).

52. J. A. Shepherd, Study of afterglow in X-ray phosphors for use on fast-framing charge-coupled device detectors. *Opt. Eng.* **36**, 3212 (1997).

53. W. J. C. Koppert, M. M. A. Dietze, S. van Der Velden, J. H. L. Steenbergen, H. W. A. M. de Jong, A comparative study of NaI(Tl), $CeBr_3$, and CZT for use in a real-time simultaneous nuclear and

fluoroscopic dual-layer detector. *Phys. Med. Biol.* **64**, 135012 (2019).

54. V. V. Nagarkar, S. Miller, B. Singh, S. Thacker, V. Gaysinskiy, B. W. Meller, H. B. Barber, D. Wilson, Development of microcolumnar $LaBr_3$:Ce scintillator. *Penetrating Radiat. Syst. Appl. X* **7450**, 745006 (2009).

55. S. Cheng, M. Nikl, A. Beitlerova, R. Kucerkova, X. Du, G. Niu, Y. Jia, J. Tang, G. Ren, Y. Wu, Ultrabright and highly efficient all-inorganic zero-dimensional perovskite scintillators. *Adv. Opt. Mater.* **9**, 2100460 (2021).

56. S. Cheng, A. Beitlerova, R. Kucerkova, E. Mihokova, M. Nikl, Z. Zhou, G. Ren, Y. Wu, Non-hygroscopic, self-absorption free, and efficient 1D $CsCu_2I_3$ perovskite single crystal for radiation detection. *ACS Appl. Mater. Interfaces* **13**, 12198–12202 (2021).

57. Y. Wei, Z. Cheng, J. Lin, An overview on enhancing the stability of lead halide perovskite quantum dots and their applications in phosphor-converted LEDs. *Chem. Soc. Rev.* **48**, 310–350 (2019).
